# Bidirectional genetic overlap between autism spectrum disorder and cognitive traits

**DOI:** 10.1038/s41398-023-02563-7

**Published:** 2023-09-14

**Authors:** Sigrun Hope, Alexey A. Shadrin, Aihua Lin, Shahram Bahrami, Linn Rødevand, Oleksandr Frei, Saira J. Hübenette, Weiqiu Cheng, Guy Hindley, Heidi Nag, Line Ulstein, Magdalena Efrim-Budisteanu, Kevin O’Connell, Anders M. Dale, Srdjan Djurovic, Terje Nærland, Ole A. Andreassen

**Affiliations:** 1 K.G. Jebsen Centre for Neurodevelopmental Disorders, Institute of Clinical Medicine, University of Oslo, Oslo, Norway; 2 Department of Neurohabilitation, Oslo University Hospital, Oslo, Norway; 3 NevSom, Department of Rare Disorders and Disabilities, Oslo University Hospital, Oslo, Norway; 4 NORMENT, Institute of Clinical Medicine, University of Oslo and Division of Mental Health and Addiction, Oslo University Hospital, Oslo, Norway; 5 Center for Bioinformatics, Department of Informatics, University of Oslo, Oslo, Norway; 6 Institute of Psychiatry, Psychology and Neuroscience, King’s College London, London, UK; 7 Frambu Resource Centre for Rare Disorders, Siggerud, Norway; 8 Haukeland University Hospital, Bergen, Norway; 9 Prof. Dr. Alex Obregia Clinical Hospital of Psychiatry, Bucharest, Romania; 10 “Victor Babes”, Național Institute of Pathology, Bucharest, Romania; 11 Department of Radiology, University of California, San Diego, La Jolla, CA USA; 12 Department of Cognitive Sciences, University of California, San Diego, La Jolla, CA USA; 13 Department of Neurosciences, University of California, San Diego, La Jolla, CA USA; 14 Department of Medical Genetics, Oslo University Hospital, Oslo, Norway; 15 NORMENT, Department of Clinical Science, University of Bergen, Bergen, Norway

**Keywords:** Clinical genetics, Genetics, Autism spectrum disorders

## Abstract

Autism spectrum disorder (ASD) is a highly heritable condition with a large variation in cognitive function. Here we investigated the shared genetic architecture between cognitive traits (intelligence (INT) and educational attainment (EDU)), and risk loci jointly associated with ASD and the cognitive traits. We analyzed data from genome-wide association studies (GWAS) of INT (*n* = 269,867), EDU (*n* = 766,345) and ASD (cases *n* = 18,381, controls *n* = 27,969). We used the bivariate causal mixture model (MiXeR) to estimate the total number of shared genetic variants, local analysis of co-variant annotation (LAVA) to estimate local genetic correlations, conditional false discovery rate (cond/conjFDR) to identify specific overlapping loci. The MiXeR analyses showed that 12.7k genetic variants are associated with ASD, of which 12.0k variants are shared with EDU, and 11.1k are shared with INT with both positive and negative relationships within overlapping variants. The majority (59–68%) of estimated shared loci have concordant effect directions, with a positive, albeit modest, genetic correlation between ASD and EDU (r_g_ = 0.21, *p* = 2e−13) and INT (r_g_ = 0.22, *p* = 4e−12). We discovered 43 loci jointly associated with ASD and cognitive traits (conjFDR<0.05), of which 27 were novel for ASD. Functional analysis revealed significant differential expression of candidate genes in the cerebellum and frontal cortex. To conclude, we quantified the genetic architecture shared between ASD and cognitive traits, demonstrated mixed effect directions, and identified the associated genetic loci and molecular pathways. The findings suggest that common genetic risk factors for ASD can underlie both better and worse cognitive functioning across the ASD spectrum, with different underlying biology.

## Introduction

Autism spectrum disorder (ASD) is a neurodevelopmental disorder characterized by difficulties in social communication and interaction as well as restrictive, repetitive patterns of behavior, interest or activities [1]. Recent studies have shown that the prevalence of ASD is 1–2% [2]. There is a large heterogeneity in cognitive functioning in ASD; with severe forms having poor cognitive functioning while others across the spectrum have better and quite extraordinary cognitive skills [3]. These large differences in cognitive ability are important for outcome [4], but the biological underpinnings for this mixed pattern of cognitive performance in ASD is not yet fully understood. Further, there is also a notion that cognitive characteristics of ASD are not necessarily deficits, but could be regarded as normal human variation [5].

The pathogenesis of ASD is considered to originate from complex interactions between environmental [6] and genetic factors, with an estimated heritability of ~80% [7]. Previous studies have shown a heterogeneous genetic architecture, with contributions from both common and rare genetic variants [8, 9]. Several common genetic variants have been discovered for ASD. The largest genome-wide association study (GWAS) of ASD to date included *n* = 18,381 cases and *n* = 27,969 controls and identified five genome-wide-significant loci [10]. By leveraging the association between ASD and three other phenotypes (schizophrenia, major depression, and educational attainment (EDU)), seven additional loci were identified [10]. However, individually these common variants have small effects, and collectively explain a small portion of the overall liability, leaving a large fraction of the heritability undiscovered [11]. Meanwhile, recent statistical tools have enabled the calculation of an individual’s genetic risk for ASD using polygenic risk scores (PGRS), which may have relevance for clinical research [12] and show promise for clinical utility in the future [13].

Intelligence and EDU are highly heritable traits which are major determinants of human health and well-being [14, 15]. Furthermore, there is phenotypic linkage between ASD and IQ/EDU and evidence of potential shared genetics [10]. Common genetic factors underlying variation in INT are also overlapping with those associated with brain volumes [16]. Thus, it is likely that common variants may relate to both the large variation in cognitive function, as well as with the large variation in brain volumes that characterize ASD [17]. Mean brain size is, however, often enlarged [18], a trait that associates with high INT [19]. Furthermore, the frontal cortex and cerebellum have been implicated in ASD pathology [20] with a tendency of large frontal lobes associated with small cerebellar volumes [21].

Recent studies suggest that 35% of ASD patients have an intellectual disability [2]. Among these patients, more than 500 rare pathogenic mutations have been discovered [22]. However, studies on rare variants may have been biased towards inclusion of patients with intellectual disability and not high-functioning ASD, which could explain why they have not offered insights into mechanisms underlying the associations between ASD and high INT [22, 23]. On the other hand, there are indications that high-functioning ASD may have been overrepresented in GWASs [23, 24], which have shown a positive genetic correlation (r_g_) between ASD and cognitive abilities [10, 25], with r_g_ = 0.2–0.3 [10, 26]. This is intriguing given that about one third of ASD children experience developmental autistic regression [27, 28] and about one third have intellectual disability [2]. Further, adults with ASD have increased risk of early onset dementia [29]. Thus, despite the overall positive genetic r_g_, between ASD and high INT, there are likely variants with an opposite effect on ASD and INT as well.

We have previously reported large polygenic overlaps despite low genetic correlation in mental disorders such as schizophrenia, ADHD and depression [30–32] by using the statistical tool bivariate causal mixture model (MiXeR) [33]. This method allows for estimating a total number of shared genetic variants, irrespective of genetic correlations between traits [33]. As such, it allows for the detection of a mixture of effect directions that would otherwise be missed with methods such as Linkage disequilibrium score regression (LDSR) [34]. Furthermore, the MiXeR results can be followed up with analysis to identify the genetic risk variants jointly associated with two traits, using conditional and conjunctional false discovery rate (condFDR/conjFDR) which increases the statistical power compared to the standard GWAS approach [33, 35]. By analyzing the molecular function of overlapping genes [36], it is possible to shed light on mechanisms underlying both high and low cognitive performance in ASD. Furthermore, while INT and EDU traits are both related to cognitive function, they have somewhat different genetic architecture [37], and seem to be associated with different characteristics among patients with ASD [38]. Thus, it is relevant to include both INT and EDU when investigating overlapping genetic architecture between ASD and cognitive traits.

Here, we took advantage of recent large GWAS data to determine the degree of overlapping genetic architecture between ASD and cognitive traits (INT and EDU) by applying MiXeR method. Second, we identified risk loci shared between ASD and the cognitive traits using the cond/conjFDR method. Third, we applied FUMA to annotate the identified loci to determine tissue expression and molecular functions of shared risk variants for ASD and cognitive traits [39].

## Methods

### Study participants

We obtained GWAS results in the form of summary statistics (*p* values and z-scores) for the relevant phenotypes [10, 40, 41] (Table 1). Data on autism spectrum disorder (ASD) were acquired from the Psychiatric Genomics Consortium (PGC) [10]. The dataset was a meta-analysis of the population-based iPSYCH project [42] and five family-based trio samples of European ancestry (*n* = 5305) [43], including a total of 18,381 ASD cases, and 27,969 controls.

**Table 1:** GWAS characteristics.

Sample	Sample size (*N*)	Age group	Reference
ASD	46,350 (ASD = 18,381, CON = 27,969)	Adults and children	Grove et al., 2019
INT	269,867	Adults and children	Savage et al., 2018
EDU	766,345	Adults	Lee et al., 2018

*ASD* autism spectrum disorder, *INT* intelligence, *EDU* educational attainment.

General Intelligence was based on data from 269,867 individuals across 14 cohorts, primarily consisting of data from the UK Biobank (*n* = 195,653) [41]. These studies assessed INT using various cognitive tests and were all operationalized to a *general intelligence* factor (g-factor). In the majority of cohorts, the g-factor was based on results on 13 different cognitive tests that required verbal and mathematical reasoning (http://biobank.ctsu.ox.ac.uk/crystal/field.cgi?id=20016) [41]. The included GWAS data from UK biobank are mainly from individuals of European descent [44].

Educational attainment (EDU) is measured as the number of years of completed schooling [31]. The GWAS data for EDU used in our analysis includes public available summary statistic from a meta-analysis of data from the Social Science Genetic Association Consortium (SSGAC), with a sample size of 766,345 individuals after excluding data from 23andMe [15]. The meta-analysis was performed using an inverse-weighted fixed effects model implemented in the METAL software (http://csg.sph.umich.edu/abecasis/metal/), of 71 quality-controlled cohort-level results files. The included GWAS data are restricted to individuals of European descent.

### Statistical analysis

We applied MiXeR v1.3 [33] to quantify polygenic overlap between ASD and cognitive traits irrespective of genetic correlation using GWAS summary statistics. This method estimates the total number of shared and trait-specific ‘causal’ SNPs and SNP-based heritability (h^2^_snp_) for each trait, based on the distribution of z-scores and detailed modeling of LD structure. Polygenicity estimates included the number of ‘causal’ variants required to explain 90% of h^2^_snp_ to prevent extrapolating model parameters into variants with infinitesimally small effects. Results were presented as Venn diagrams displaying the proportion of trait-specific and shared ‘causal’ SNPs. Dice coefficient as calculated by MiXeR was used to estimate the similarity between genetic architecture of two phenotypes. Model fit was evaluated based on predicted versus observed conditional quantile-quantile (Q–Q) plots, the Akaike Information Criterion (AIC) and log-likelihood plots (Supplementary Methods). A positive AIC indicates adequate discrimination between modeled fit and the comparative model. A negative AIC indicates inadequate discrimination between modeled fit and the comparative model.

We next applied the conditional(cond)/conjunctional(conj)FDR method, which leverages polygenic overlap between two traits to boost statistical power to identify loci associated with a single trait (condFDR) and loci jointly associated with two traits (conjFDR) [35]. Cross-trait enrichment of SNP associations between ASD and each cognitive trait, and vice versa, was visualized using conditional Q–Q plots. The condFDR value of each SNP was computed for ASD conditional on cognitive traits and vice versa. CondFDR represents the probability that a SNP is not associated with the primary trait given that the *p*-values in the primary and conditional trait are as small as or smaller than the observed *p*-values. Next, the conjFDR value for each SNP was calculated as the maximum of the two condFDR values (i.e., ASD conditional on INT and vice versa). This represents a conservative estimate of the FDR for the association between each SNP with both traits. SNPs with a condFDR <0.01 or conjFDR<0.05 were assigned statistical significance. Since the complex correlations in regions with intricate linkage disequilibrium [45] can bias FDR estimation, all cond/conjFDR analyses were performed after excluding the following SNPs regions from the FDR fitting procedures: the extended major histocompatibility complex (MHC) region (chr6: 25119106-33854733), the 8p23.1 region (chr8: 7242715-12483982) and the MAPT region (chr17: 40000000-47000000). However, they were not excluded from our discovery analysis. All chromosome locations are derived from genome build hg19. We further evaluated the directional effects of the shared loci by comparing their z-scores from original GWAS. We also identified previously reported GWAS associations in the NHGRI-EBI catalog [46] overlapping with the identified loci. For more details about the statistical tools, see Supplementary Methods and the original publications [33, 47].

### Genetic loci definition and effect direction

We defined independent genetic loci according to the FUMA protocol [39]. We evaluated the directional effects of shared loci by comparing *z* scores from the respective GWAS summary statistics.

### Genome-wide and local genetic correlations

Genome-wide genetic correlation (r_g_) was estimated using linkage disequilibrium score regression (LDSR) [48]. Local heritabilities and local genetic correlations within shared loci identified in conjFDR analyses were calculated using local analysis of co-variant annotation (LAVA) [49]. See Supplementary Methods for more details.

### Functional annotation

We functionally annotated all candidate SNPs in the genomic loci with a conjFDR value < 0.1 having an LD *r*^2^ ≥ 0.6 with one of the independent significant SNPs, using FUMA SNP2GENE [39]. We linked lead SNPs to genes using three gene-mapping strategies: (1) positional mapping to align SNPs to genes based on their physical proximity, (2) expression quantitative trait locus (eQTL) mapping to match cis-eQTL SNPs to genes whose expression is associated with allelic variation at the SNP level, and (3) chromatin interaction mapping to link SNPs to genes based on three-dimensional DNA–DNA interactions between each SNP’s genomic region and nearby or distant genes. All gene-mapping strategies were limited to brain tissues. Finally, we queried SNPs for known QTLs in brain tissues using the GTEx portal (GTEx, version 8) [50]. If the gene annotation of a specific SNP was marked as ‘NA’, we search for information in the dbSNP database. We investigated whether genes mapped to SNPs in the shared loci were overrepresented in gene-sets and biological pathways using FUMA GENE2FUNC [39] (see Supplementary Methods).

## Results

### Shared genetic architecture (MiXeR)

**Fig. 1:**
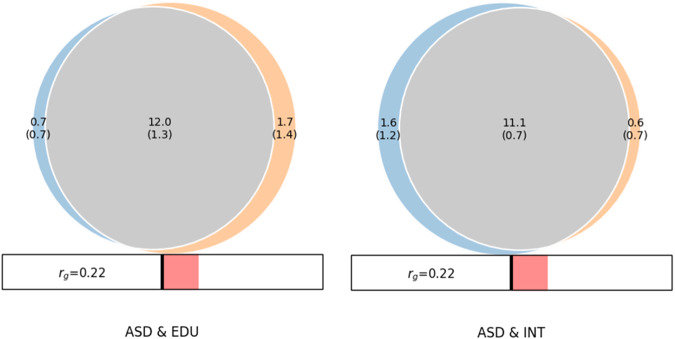
MiXeR-modeled genome-wide genetic overlap between autism spectrum disorder (ASD), educational attainment (EDU) and intelligence (INT). Venn diagrams from MiXer analyses shows the number of shared and trait-specific “causal” genetic variants in thousands for ASD & EDU and ASD & INT. The MiXeR estimated DICE coefficient for ASD & EDU was 0.90 and for ASD & INT it was 0.91. Both analyses had positive AIC values when comparing modeled estimates to minimum possible overlap but negative compared to maximum possible overlap, indicating that the estimates may underestimate genetic overlap. Rg: MiXeR estimated genome-wide genetic correlation.

*MiXeR* revealed substantial amount of shared ‘causal’ variants between ASD&INT and ASD&EDU. As shown in the Venn diagram (Fig. 1), the estimated number of shared ‘causal’ variants between ASD and INT was 11.1k (SD = 0.7k), with 1.6k (1.2k) unique ASD variants and 0.6k (0.7k) unique INT variants. The Dice coefficient was 0.91 for variants shared between ASD and INT (Table S15). MiXeR estimated 12.0k (1.3k) shared ‘causal’ variants between ASD and EDU, with 0.7k (0.7k) unique ASD variants and 1.7k (1.4k) unique EDU variants. The Dice coefficient was 0.90 for variants shared between ASD and EDU (Table S15). The proportion of shared ‘causal’ variants with concordant effects for ASD&INT was 0.58 (SD = 0.004) and 0.58 (SD = 0.005) for ASD&EDU.

### Enrichment

**Fig. 2:**
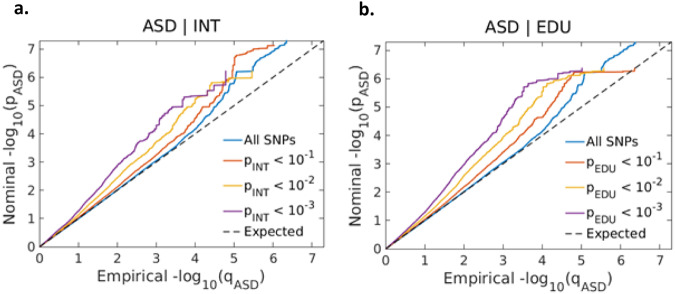
Conditional Q–Q plots. Conditional QQ plots of observed versus expected −log10 *p*-values in the primary trait (ASD) as a function of significance of genetic association with the secondary traits intelligence (**a**) and educational attainment (**b**) at the level of *p* ≤ 0.1 (red lines), *p* ≤ 0.01 (yellow lines) and *p* ≤ 0.001 (purple lines). Blue lines indicate all SNPs. Black dotted line is the expected Q–Q plot under the null hypothesis (no SNPs associated with the trait).

In the conditional Q–Q plots, we observed SNP enrichment for ASD as a function of the significance of SNP associations with EDU (Fig. 2a) and INT (Fig. 2b). The reverse conditional Q–Q plots also demonstrate consistent enrichment in ASD given associations with INT and EDU, indicating polygenic overlap between the phenotypes (Fig. S1a, S1b).

Log-likelihood plots are shown in Figs. S1a and S1b. The AIC values (Table S15) were positive when comparing modeled estimates to minimum overlap, but negative compared to maximum overlap for both ASD/INT and ASD/EDU analysis. This indicates that the MiXeR-predicted overlap is not distinguishable from maximum possible overlap, suggesting caution in interpreting the estimates from MiXeR. ASD and INT have LDSR-based genome-wide genetic correlation of r_g_ = 0.22 (SD = 0.032, *p* = 4.60e−12) and MiXeR-estimated genetic correlation of shared variants of ρβ = 0.24 (SD = 0.01). For ASD and EDU, those values are respectively r_g_ = 0.21 (SD = 0.028, *p* = 2.17e−13) and ρβ = 0.25 (SD = 0.02). This pattern of extensive genetic overlap but weak genetic correlation is indicative of mixed effect directions, supported by the MiXeR-estimated proportion of shared ‘causal’ genetic variants with concordant effects of 0.58 for both ASD&INT and ASD&EDU.

### Identification of shared genetic loci (cond/conjFDR)

#### CondFDR

We leveraged this pleiotropic enrichment using condFDR analysis and re-ranked the ASD SNPs conditional on their association with EDU or INT, and vice versa. At condFDR <0.01, there were 9 loci associated with ASD conditional on INT (Table S1), of which two loci were not found in the original ASD GWAS (Table S1). We identified 12 loci associated with ASD conditional on EDU (Table S2), of which four were not in identified the original ASD GWAS (Table S2).

#### ConjFDR

**Fig. 3:**
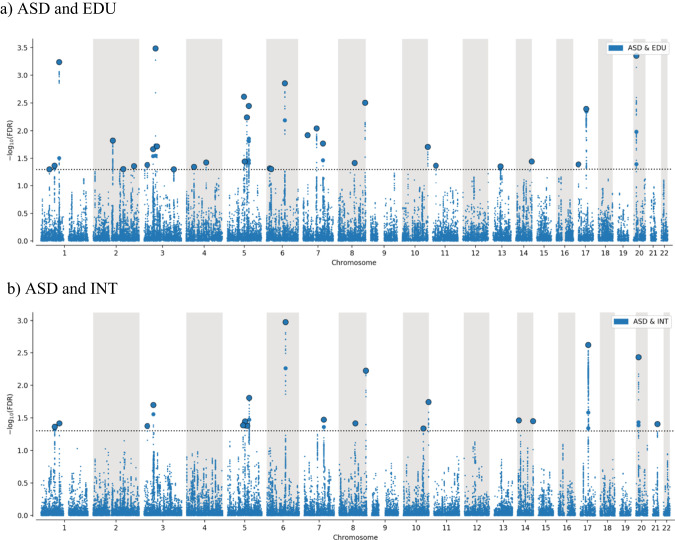
Manhattan plots showing common genetic variants jointly associated with autism (ASD) and cognitive traits. The plots show common genetic variants jointly associated with ASD and intelligence (**a**) and ASD and educational attainment (**b**) with the –log10 transformed conjFDR values for each SNP on the *y*-axis and chromosomal positions on the *x*-axis. The black dotted horizontal line represents the threshold for significant shared associations (conjFDR <0.05, i.e. –log10 (conjFDR >1.3)). Independent lead SNPs are encircled in black.

The conjFDR Manhattan plots are shown in Fig. 3a, b. At conjFDR < 0.05, we detected 19 genetic loci jointly associated with ASD and INT (Table S3), and among them, 11 are unique for ASD and INT. We detected 32 distinct genetic loci jointly associated with ASD and EDU (Table S4), of which 24 are unique for ASD and EDU. Eight loci were common for both ASD and EDU and ASD and INT, yielding a total of 43 distinct loci at conjFDR < 0.05. Of these SNPs, 18 were intronic, 13 intergenic, 11 non-coding RNA intronic and 1 exonic (see Tables S3 and S4).

#### Evaluation of allelic effect directions

Loci were either concordant or discordant as denoted by the sign of the effect, and 68% (13/19) of the shared loci between ASD and INT had concordant allelic effect directions (Table S3) and 59% (19/32) of the shared loci between ASD and EDU possessed concordant allelic effect directions (Table S4).

#### Local genetic correlations

LAVA analysis of 19 loci shared between ASD and INT revealed three loci (2q12.1, 5q22.3 and 14q32.33) with significant local heritabilities (*p* < 0.05/19) in both ASD and INT and nominally significant local genetic correlation (*p* < 0.05) (marked with green in Table S3), all being positive. For 32 loci shared between ASD and EDU, LAVA identified five loci (6q16.1, 6p21.32, 7p15.3, 14q32.33 and 17q21.31) with significant (*p* < 0.05/32) local heritabilities in both ASD and EDU and significant (*p* < 0.05) genetic correlation between them (marked with green in Table S4), four out of these five loci were positively correlated while one locus had negative correlation.

#### Novel ASD loci

As seen in Table S3, 11 of 19 the lead SNPS jointly associated with ASD and INT at conjFDR <0.05, were not identified in the original ASD GWAS [10], and 21 of the 32 loci jointly associated ASD and EDU were also novel (Table S4). Five of these loci were overlapping both with EDU and INT, which yielded a total of 27 novel ASD loci (Table 2).

**Table 2:** Novel shared SNP’s between ASD and INT, and ASD and EDU found through cond/conjFDR.

Chr	Min-max BPs	Lead SNPs	conjFDR	ASD		Trait (INT/EDU)			
				Z-score	*p*-value	Z-score	*p*-value	Concordant	Overlapping
*ASD and INT*									
3	16843737-16879208	rs7625233	0.042	3.9	1.14E−04	−4.88	1.07E−06	No	Yes
3	48564209-50239012	rs73073015	0.020	4.1	3.51E−05	6.28	3.43E−10	Yes	Yes
5	81261923-81679914	rs73134709	0.041	−3.9	9.58E−05	−3.86	1.16E−04	Yes	No
5	92488009-92574385	rs4242244	0.036	−3.9	8.64E−05	−5.48	4.16E−08	Yes	Yes
5*	113837198-113995764	rs414517	0.016	−4.23	2.30E−05	−4.25	2.18E−05	Yes	No
8	87754626-87783335	rs1982564	0.038	3.90	9.62E−05	−4.01	6.14E−05	No	Yes
10	106563924-106830537	rs6584649	0.046	−3.82	1.33E−04	3.88	1.05E−04	No	No
10	133729181-133815530	rs34473884	0.018	4.17	3.03E−05	5.26	1.48E−07	Yes	Yes
14	29396922-29677464	rs140802584	0.034	4.02	5.87E−05	−3.93	8.42E−05	No	No
17	43463493-44865603	rs7207582	0.002	4.71	2.44E−06	−4.91	9.22E−07	No	No
21	40553845-40741068	rs2249666	0.039	3.89	9.89E−05	4.06	4.99E−05	Yes	No
*ASD and EDU*									
1	45797505-46021556	rs12049503	0.050	3.77	1.63E−04	4.10	4.12E−05	Yes	No
2*	104056454-104387855	rs6543224	0.015	4.26	2.05E−05	5.01	5.32E−07	Yes	No
2	159340038-159553686	rs3771643	0.049	3.80	1.46E−04	3.97	7.29E−05	Yes	No
2	215361613-215406125	rs12467438	0.044	−3.84	1.25E−04	4.28	1.85E−05	NO	No
3	16843737-16879208	rs7625233	0.042	3.86	1.14E−04	−6.37	1.83E−10	No	Yes
3	48564209-50239012	rs73073015	0.021	4.14	3.51E−05	7.25	4.14E−13	Yes	Yes
3	70252572-70291268	rs73116288	0.019	4.18	2.93E−05	4.53	5.89E−06	Yes	No
3	157829953-158284861	rs7630176	0.050	−3.77	1.63E−04	4.13	3.58E−05	No	No
4	105319081-105414222	rs7665487	0.037	3.91	9.27E−05	−4.28	1.84E−05	No	No
5	87792844-87932809	rs4916723	0.002	4.76	1.92E−06	−7.09	1.32E−12	No	No
5	92488009-92574385	rs4242244	0.036	−3.93	8.64E−05	−5.04	4.75E−07	Yes	Yes
5	113788755-113995764	rs13188074	0.004	4.67	3.04E−06	5.30	1.18E−07	Yes	No
6	19211776-19358341	rs7762189	0.048	3.79	1.51E−04	−4.60	4.25E−06	No	No
6	26341301-26341301	rs9467715	0.049	−3.78	1.60E−04	−5.42	5.98E−08	Yes	No
7*	24526039-24536700	rs6461809	0.012	4.33	1.48E−05	6.04	1.55E−09	Yes	No
8	87754626-87783335	rs1982564	0.038	3.90	9.62E−05	−5.46	4.75E−08	No	Yes
10	133729181-133815530	rs34473884	0.020	4.17	3.03E−05	7.40	1.32E−13	Yes	Yes
11	17804998-17852452	rs2237944	0.042	3.85	1.18E−04	4.69	2.69E−06	Yes	No
13	58746132-59167198	rs77146055	0.044	3.83	1.26E−04	−4.02	5.90E−05	No	No
17	2295405-2296014	rs2447091	0.041	3.87	1.09E−04	−4.68	2.89E−06	No	No
17*	43463493-44865603	rs55915917	0.004	4.64	3.55E−06	−8.39	4.93E−17	No	No

*Chr* Chromosome, *Min-max BPs* Minimum-Maximum Base Pairs, *Lead SNP* Single Nucleotide Polymorphism within a locus having the lowest *P*-value, *conjFDR* Conjunctional False Discovery Rate, *ASD* Autism Spectrum Disorder, *INT* Intelligence, *EDU* Educational attainment, *Overlapping* overlapping SNP’s between INT and EDU.

*Loci with significant local genetic correlation.

#### Functional annotation (FUMA SNP2GENE)

We did functional annotation of all SNPs with a conjFDR value < 0.1 within loci shared between ASD & INT and ASD & EDU, which resulted in 2356 candidate SNPs jointly associated with ASD and INT (Table S5) and 1782 SNPs candidate SNPs jointly associated with ASD and EDU (see Table S6).

#### Gene-mapping

By using three different methods (positional, eQTL, and chromatin interaction) we mapped 104 genes from candidate SNPs within loci shared between ASD and INT (see Table S7) and 132 genes for ASD and EDU (see Table S8). Of these, there were 10 genes that were credible i.e., implicated by all three mapping strategies in analysis of ASD and EDU and all of these were also credible in analysis of ASD and INT, resulting in 16 credible mapped genes all together (see Fig. S9 and Table S16).

### Gene-set enrichment and molecular function analysis (FUMA GENE2FUNC)

#### Gene expression in different tissues

Heatmaps of all genes annotated to candidate SNPs are shown in Fig. S4a (ASD and EDU) and Fig. S5a (ASD and INT). Candidate genes from ASD and EDU had significantly upregulated differentially expressed genes (DEGs) in four of 54 different tissues, namely brain cortex, frontal cortex, brain cerebellum and cerebellar hemisphere (Fig. S4b) and candidate genes from ASD and INT had significant upregulated DEGs two tissues: cerebellum and cerebellar hemisphere (Fig. S5b).

#### Gene expression during brain development periods

Candidate genes tended to have upregulated expression during early prenatal period and late infancy (Figs. S3c and S4c) but these differences were not significant.

#### Gene set enrichments

GO biological processes molecular function (Tables S9 and S10): Enrichment was found in 43 different gene sets, including positive regulation of central nervous system development, midbrain development, neuronal differentiation, synaptic signaling, neuron death, gliogenesis, astrocyte development, mitochondrion organization, synapse plasticity and more general pathways as inositol phosphate and response to reactive oxygen species,

#### Transcription factors

Candidate genes were enriched in the pathways of 100 transcription factors, of them HIF1 (hypoxia inducible factor 1), NFR1 (nuclear respiratory factor 1) and vitamin D receptor.

#### Immunologic signatures

Candidate genes were enrichments in 23 immune related gene sets for ASD and EDU, among them, Interleukin-2 and Interleukin-10 pathways, Macrophage Stimulating 1 (MSP1) pathway, EBNA1 anticorrelated, and development of regulatory T cells (Tregs).

#### GWAS gene sets

As seen in Tables S9 and S10, enrichment was seen in 100 different gene sets including ASD related social behaviors (attendance at social groups, helping behavior), cognitive function, mental/neurologic traits (short sleep, alcohol abuse, mood instability, schizophrenia, depression, neuroticism, intracranial volume, neurodegenerative diseases) and somatic traits (inflammatory bowel diseases, cardiovascular measures, lung function/pulmonary fibrosis, endocrine measures).

#### FUMA (GENE2FUNC) of concordant loci (Figs. S5–6 and Tables S11 and S13)

Heatmaps showing the tissue expressions of each gene in the concordant gene sets (ASD/EDU and ASD/INT) are shown in Figs. S5a–S6a. For ASD/INT, expression analyses showed that concordant genes were significantly differently expressed (DEGs) in 13 tissues, with highest DEGs in frontal cortex (Fig. S5b). Similar results were found for ASD/EDU, were DEGs were significantly less expressed in amygdala, hippocampus, basal ganglia, and substantia nigra. Highest upregulation (non-significant) was found in brain frontal cortex and cerebellum (Fig. S6b). Similar enrichment analyses as for the total gene sets were performed for concordant genes and showed that they were enriched in gene sets for extremely high intelligence, social traits (attending social groups and helping behavior), psychiatric disorders, inflammatory bowel diseases and immunological signatures (Tables S11 and S13). FUMA analyses of the 6 credible genes mapped from concordant loci (*NCKIPSD, CCDC36, IP6K2, PRKAR2A, QRICH1, CCDC71*) showed that they were enriched in pathways for inflammatory diseases and blood protein levels (Fig. S9a and Table S16).

*FUMA GENE2FUNC of discordant loci* (Figs. S7–S8 and Tables S12 and S14) showed that they were significantly upregulated (DEGs) in the cerebellum and cerebellar hemisphere (Figs. S7b and S8b). Discordant genes were enriched in several gene sets, including neurodegenerative diseases (incl. Alzheimer’s disease and Parkinson’s disease), chronic pain, alcohol use disorder and craniofacial macrosomia (small head and face) (Tables S12 and S14). For the credible mapped discordant genes (*MAPT, CRHR1, WNT3, KANSL1, ARL17B, SPPL2C, LRRC37A, ARHGAP27, PLEKHM1,* and *STH*) we found trends of similar enrichments as the total set of discordant genes (Fig. S9b and Table S16).

## Discussion

The main finding of the current study is an extensive genetic overlap between ASD and the cognitive traits INT and EDU with a mixture of positive and negative effect directions of the overlapping genetic loci. We identified 43 loci jointly associated with ASD and INT or EDU, of which 27 were novel for ASD. The results provide insights into putative overlapping molecular mechanisms. By dissecting the overlapping genetic architecture and quantifying the shared and unique genetic factors for ASD versus cognitive traits beyond genetic correlations, we show that common genetic variants can underlie both better and worse cognitive functioning across the ASD spectrum.

The current findings of bidirectional genetic overlap between ASD and cognitive traits INT and EDU, as revealed with the MiXeR method, has not been shown before. The genetic overlap estimated by Dice coefficient was 0.90–0.91 which is substantial, taking into account the relatively low genetic correlation we found between ASD and INT (r_g_ = 0.22), in line with previous findings [10]. It is noteworthy that the genetic correlation is only present if the bulk of variants associated with both ASD and INT or EDU have consistent direction of effects (concordant or discordant) but not mixed [51]. Among the 43 loci shared between ASD and EDU or INT revealed by conjFDR, *n* = 27 (63%) had concordant effect directions with INT and EDU. Thus, the main fraction of common variants shared with ASD is associated with higher INT and EDU. These variants may shed light on mechanisms underlying better cognition in ASD patients [10, 52, 53] and provide support for high functioning ASD as a “neurodiversity” rather than a disorder [5].

A high genetic overlap between ASD and cognitive traits INT and EDU is consistent with genetic overlap between INT and EDU and other mental disorders, such as schizophrenia (SCZ) [30, 54], bipolar disorder (BP) [30], major depression (MD) [32] and attention deficit hyperactivity disorder (ADHD) [31], although the overlap between ASD and INT is larger than between INT and SCZ, BP, ADHD and MD [30–32]. However, the overall concordant effect direction with INT contrasts findings in SCZ and ADHD where the majority of variants shared with INT are associated with poorer cognitive performance [30, 31]. The results also differ from MD and BP which have a more balanced mixture of directional effects among the loci shared with INT [30, 32]. A potential clinical implication of the current result is to improve ASD polygenic scores to stratify ASD according to genetic variants differentiating between reduced and improved cognitive abilities. This can help to target interventions against risk of autistic regression and dementia among adults, in a precision medicine approach.

Analyses of brain tissue expression of all candidate genes, including both concordant and discordant showed that they are significantly upregulated in two brain tissues in frontal cortex and cerebellum, which is in line with a recent meta-analysis of post-mortem studies in ASD [20]. In recent years the interest in cerebellum’s role in language and social behavior has increased [55] and it has emerged as key for ASD pathology [56, 57]. The increased expression in cerebellum was only significant for discordant genes. This seems in line with the association between motor impairments and cognitive impairments in ASD [58]. Concordant genes did not have significantly upregulated DEGs in any of the brain tissues investigated, suggesting that they are not especially important for these brain regions. Associated genes were, however, enriched in the pathways for midbrain development, a region not included in the tissue analysis. Still, its relevance in ASD is supported by a genetic overlap between determinants of midbrain volume and ASD [59], and the concordant gene *RHOA* has been targeted for improved learning and memory in ASD animal models [60]. As expected, associated genes were enriched in several gene sets important for neurodevelopment, and with gene sets reflecting social function, as e.g., helping behavior and participating in social groups. These enrichments suggest that the associated genes are of relevance for ASD.

Genes associated with concordant loci were enriched in a pathway for extremely high INT [61], and included the gene for creatine kinase, brain type (*CKB*). This seems in line with that creatine has been suggested as a cognitive enhancer [62], The concordant genes were also enriched in 23 immune pathways and in inflammatory bowel diseases. One of these genes was *MST1*, which is found in the high intelligence-pathway and plays a role in autoimmunity [63]. This support the involvement of inflammation in ASD [64] and is consistent with cytokines as positive modulators of cognitive function [65, 66]. Concordant genes were also enriched in the pathway of vitamin D receptor, which may be relevant for the association between ASD and cognitive function [67, 68].

Discordant credible genes were enriched in three types of GWAS phenotypes, mental disorders, neurodegenerative diseases and somatic traits. Of these, the enrichment in neurodegenerative diseases as Alzheimer’s and Parkinson’s is of interest since the variants could possibly be involved in mechanisms underlying autistic regression in children and of increased risk of dementia in adults [29, 66]. Among credible genes enriched in neurodegenerative diseases are *CRHR1, KANSL1, MAPT*, and *WNT3*. *CRHR1* encodes a corticotrophin releasing hormone receptor implicated in social behavior [69, 70] and stress-induced cognitive deficits [71]. *KANSL1* has been associated with autistic traits [72] and cognitive difficulties in 17.q21.31 deletion syndrome [73]. *MAPT* encodes the tau–protein which misfolds and forms a hallmark of frontotemporal dementia and Alzheimer’s disease [74]. *WNT3* is a Wnt-signaling gene involved in neurogenesis [75], as well as in behavioral and cognitive deficits [75]. It has been suggested that the Wnt-pathway may be of importance for understanding the high phenotypical heterogeneity of ASD [76]. Together, discovery of these discordant genes could potentially improve the understanding of autistic regression and cognitive difficulties in ASD.

A limitation of our study is that the sample of UK-biobank consists mainly of persons of European ancestries. Another limitation is that the study does not include rare pathogenic variants causing ASD, as only common variants are included in the analyses. Furthermore, the results are based on a common factor for INT, which is not exactly similar with a full IQ score. Furthermore, EDU is not purely a cognitive trait, but it is also influenced by other factors, including socioeconomic status.

In conclusion, the current findings show extensive bidirectional genetic overlap between ASD and cognitive traits, with a majority of loci for ASD associated with better cognitive performance. The mixture of effect directions is in line with the large variation in cognitive abilities in ASD. Together, these findings suggest that genetic factors may underlie some of the large variation in cognitive performance in ASD, and highlight molecular mechanisms involved in the two cognitive subgroups within the ASD spectrum.

### Supplementary information

Supplementary information regarding methods

Supplementary Results, Figure S1-S9 and Table 16

Supplementary Results, Tables S1-S15

## Data Availability

Data supporting the findings of this study are openly available from an online repository or are available on request from study authors. The dataset regarding ASD is available in repositories of GWASs: ASD2019: https://www.med.unc.edu/pgc/download-results/. Please refer to Supplementary Methods for further details.

## Code availability

All codes are freely available at https://github.com/precimed and https://github.com/bulik/ldsc. Analyses were conducted in Python v3.5, Matlab R2020b. Locus definition, functional annotation, and gene-set analysis were performed using FUMA (https://fuma.ctglab.nl/).
