# Supplementary material for: Bidirectional genetic overlap between autism spectrum disorder and cognitive traits
Source: Transl Psychiatry. 2023 Sep 14;13:295. doi: 10.1038/s41398-023-02563-7 (PMC10502136; doi:10.1038/s41398-023-02563-7)
Supplement: Supplementary file 1 — Supplementary information regarding methods [file 41398_2023_2563_MOESM1_ESM.docx]

**Supplementary information**

for

**Bidirectional genetic overlap between autism spectrum disorder and cognitive traits**

Content^[[1]](#footnote-1)^

[MiXeR 1](#_Toc133343474)

[Genetic correlations, LD score regression and LAVA 3](#_Toc133343475)

[*Genome-wide genetic correlation:* 3](#_Toc133343476)

[*This* 3](#_Toc133343477)

[*Local genetic correlations (LAVA)* and local SNP heritabilities: 3](#_Toc133343478)

[Conditional False Discovery Rate 4](#_Toc133343479)

[Conditional Q-Q plots 5](#_Toc133343480)

[Detection of SNPs using conditional and conjunctional FDR 7](#_Toc133343481)

[Genomic loci definition 8](#_Toc133343482)

[Functional annotation (FUMA) 8](#_Toc133343483)

[FUMA SNP2GENE 8](#_Toc133343484)

[FUMA GENE2FUNC 10](#_Toc133343485)

[Supplementary References 10](#_Toc133343486)

# MiXeR

The MiXeR tool (<https://github.com/precimed/mixer>) applies causal mixture models to GWAS summary statistics. For each SNP, $i$, univariate MiXeR models its additive genetic effect of allele substitution,$\beta_{i}$, as a point-normal mixture, $\beta_{i}=\left( 1-\pi_{1} \right)N\left( 0,0 \right)+\pi_{1}N(0, \sigma_{\beta}^{2})$, where $\pi_{1}$ represents the proportion of non-null SNPs (`polygenicity`) and $\sigma_{\beta}^{2}$ represents variance of effect sizes of non-null SNPs (`discoverability`). Then, for each SNP, $j$, MiXeR incorporates LD information and allele frequencies for M=9,997,231 SNPs extracted from 1000 Genomes Phase3 data by LD score regression software [4], and estimate the expected probability distribution of the signed test statistic, $z_{j}=\delta_{j}+\epsilon_{j}=N\sum_{i} \sqrt{H_{i}}r_{ij}\beta_{i}+\epsilon_{j}$, where $N$ is sample size, $H_{i}$ indicates heterozygosity of i-th SNP, $r_{ij}$ indicates allelic correlation between i-th and j-th SNPs, and $\epsilon_{j}\sim N(0, \sigma_{0}^{2})$ is the residual variance. Further, the three parameters, $\pi_{1}, \sigma_{\beta}^{2}, \sigma_{0}^{2}$, are fitted by direct maximization of the likelihood function. The number of causal variants is estimated as $M\pi_{1}$, where M=9,997,231 gives the number of SNPs in the reference panel.

In the cross-trait analysis, MiXeR models additive genetic effects as a mixture of four components, representing null SNPs in both traits ($\pi_{0})$; SNPs with a specific effect on the first and on the second trait ($\pi_{1}$ and $\pi_{2}$, respectively); and SNPs with non-zero effect on both traits ($\pi_{12}$). In the last component, MiXeR models variance-covariance matrix as $\boldsymbol{\Sigma}_{\mathbf{12}}=\left[ \begin{matrix} \sigma_{1}^{2} & {\rho_{12}\sigma}_{1}\sigma_{2} \\ {\rho_{12}\sigma}_{1}\sigma_{2} & \sigma_{2}^{2} \end{matrix} \right]$ where $\rho_{12}$ indicates correlation of effect sizes within the shared component, and $\sigma_{1}^{2}$ and $\sigma_{2}^{2}$ correspond to the discoverability parameter estimated in the univariate analysis of the two traits. After fitting parameters of the model, the Dice coefficient of polygenic overlap is then calculated as $\frac{2\pi_{12}}{\pi_{1}+2\pi_{12}+\pi_{2}}$, and genetic correlation is calculated as $r_{g}=\frac{\rho_{12}\pi_{12}}{\sqrt{(\pi_{1}+\pi_{12})(\pi_{2}+\pi_{12})}}.$ Further information is available in[2].

A cut-off of 90% SNP heritability is chosen to avoid extrapolating the model parameters into SNP associations with infinitesimally small effect-sizes.

Univariate and bivariate estimates and standard errors were calculated by performing 20 iterations with 2 million randomly selected SNPs followed by random pruning at an LD threshold of r^2^=0.8, resulting in a sample of ~600K SNPs per iteration. The mean and standard error were then computed for each variable from the resulting sample of 20 iterations for each analysis.

To filter situations with insufficiently powered GWAS summary statistics, we use Akaike information criterion ($AIC=2k-2\ln L$), where $k$ is the number of free parameters in the model, $L$ is the value of the likelihood function, and $n$ is the effective number of SNPs used in optimization procedure. We calculate the difference between *AIC* for the full bivariate model, $k=3$, and AIC for the reduced bivariate model, $k=2$, due to $\pi_{12}$ being constrained to smallest or largest possible ( $\pi_{12}^{min}=r_{g}\sqrt{\pi_{1}^{u} \pi_{2}^{u}}$ and $\pi_{12}^{max}=min(\pi_{1}^{u}, \pi_{2}^{u})$, respectively). A positive value of AIC indicates that GWAS summary statistics have enough information to distinguish the custom polygenic overlap, as shown on the MiXeR Venn diagrams, versus the constrained models with minimal ($\pi_{12}^{min}$) and maximum ($\pi_{12}^{max}$) polygenic overlap. MiXeR results are presented as a Venn diagram of shared and unique polygenic components across traits.

# Genetic correlations, LD score regression and LAVA

## *Genome-wide genetic correlation:*

*This* was estimated using Linkage Disequilibrium Score Regression (LDSR) [1, 29]. This is a statistical procedure that control for overlapping samples without requiring individual genotype data. LD score regression was estimated using the Python-based package available at https://github.com/bulik/ldsc. The procedure is described in the documentation of the package (<https://github.com/bulik/ldsc/wiki/Heritability-and-Genetic-Correlation>).

## *Local genetic correlations (LAVA)* and local SNP heritabilities:

These were calculated using LAVA (Local Analysis of [co]Variant Association) [30]. Traditionally, analyses of genetic correlation (rg) consider only the average of the shared signal across the genome, and thus is not able to discover correlations confined to particular genomic regions. LAVA is a newly developed statistical tool that enables estimation of genetic correlations within specified region of the genome allowing correlations with opposing directions at different loci [30]. We performed LAVA analysis for 19 loci shared between ASD and INT and 32 loci shared between ASD and EDU as identified in corresponding conjFDR analysis. Loci boundaries were defined based of FUMA protocol as described in “Genomic loci definition” section below. We used R code and followed analysis proceure provided at https://github.com/josefin-werme/LAVA with linkage disequilibrium structure estimated based on the 1000 Genomes phase 3 European data.

# Conditional False Discovery Rate

The ‘enrichment’ seen in the conditional Q-Q plots can be directly interpreted in terms of true discovery rate (TDR = 1 – false discovery rate (FDR))[5]. More specifically, for a given p-value cutoff, the FDR is defined as

FDR(p) = π_0_F_0_(p) / F(p), [1]

where π_0_ is the proportion of null SNPs, F_0_ is the null cumulative distribution function (cdf), and F is the cdf of all SNPs, both null and non-null[6]. Here, we assume the SNP *p* values are a priori independent and identically distributed. Under the null hypothesis, F_0_ is the cdf of the uniform distribution on the unit interval [0,1], so that Eq. [1] reduces to

FDR(p) = π_0_p / F(p), [2]

The cdf F can be estimated by the empirical cdf q = N_p_ / Ν, where N_p_ is the number of SNPs with p-values < p, and N is the total number of SNPs. Replacing F by q in Eq. [2], we get

Estimated FDR(p) = π_0_p / q, [3]

which is biased upwards as an estimate of the FDR[7]. Replacing π_0_ in Equation [3] with unity gives an estimated FDR that is further biased upward;

q* = p / q, [4]

If π_0_ is close to one, which is probably true for most GWASs, the increase in bias from Eq. [3] is minimal. Therefore, the quantity 1 – p/q, is biased downward and thus a conservative estimate of the TDR. Referring to the Q-Q plots, we see that q* is equivalent to the nominal p-value divided by the empirical quantile, as defined previously. We can thus read the FDR estimate directly off the Q-Q plot as

-log_10_(q*) = log_10_(q) – log_10_(p), [5]

demonstrating that the estimated FDR is directly related to the horizontal shift of the curves in the Q-Q plots from the expected line x = y, i.e. a larger shift corresponds to a smaller FDR.

# Conditional Q-Q plots

Q-Q plots compare a nominal probability distribution against an empirical distribution. In the presence of all null relationships, nominal p-values form a straight line on a Q-Q plot when plotted against the empirical distribution. For ASD, INT and EDU risk factor SNPs and for each categorical subset (strata), -log_10_ nominal p-values were plotted against -log_10_ empirical p-values (conditional Q-Q plots). Leftward deflections of the observed distribution from the projected null line illustrate increased tail probabilities in the distribution of test statistics (z-scores) and consequently an over-abundance of low p-values compared to that expected by chance, also called ‘enrichment’.

Under large-scale testing paradigms, such as GWAS, we can calculate quantitative estimates of likely true associations from the distributions of summary statistics[6, 8]. Conditional Q-Q plots of nominal p-values from GWAS summary statistics visualizes this enrichment of statistical association relative to that expected under the global null hypothesis. The usual Q-Q curve has the nominal p value, denoted by "p", as the y-ordinate and the corresponding value of the empirical cdf, denoted by "q", as the x-ordinate. Under the global null hypothesis the theoretical distribution is uniform on the interval [0,1]. As is common in GWAS, we instead plot -log_10_ p against -log_10_ q to emphasize tail probabilities of the theoretical and empirical distributions. Therefore, genetic enrichment is illustrated with a leftward shift in the Q-Q curve, corresponding to a larger fraction of SNPs with nominal -log_10_ p-value greater than or equal to a given threshold. Conditional Q-Q plots are constructed by creating subsets of SNPs based on levels of an auxiliary measure for each SNP, and computing Q-Q plots separately for each level. If SNP enrichment is captured by variation in the auxiliary measure, this is expressed as successive leftward deflections in a conditional Q-Q plot as levels of the auxiliary measure increase. We constructed conditional Q-Q plots of empirical quantiles of nominal -log_10_ values for SNP association for all SNPs, and for subsets (strata) of SNPs determined by the nominal p-values of their association with the conditional phenotypes, and vice versa. In particular, we computed the empirical cumulative distribution (cdf) of nominal p-values for a given phenotype for all SNPs and for SNPs with significance levels below the indicated cut-offs for the conditional phenotypes (-log_10_(p) ≥ 1, -log_10_(p) ≥ 2, -log_10_(p) ≥ 3 corresponding to p < 0.1, p < 0.01, p < 0.001 respectively). The nominal p-values (–log_10_(p)) are plotted on the y-axis, and the empirical quantiles (–log_10_(q), where q=1-cdf(p)) are plotted on the x-axis. To assess for polygenic effects below the standard GWAS significance threshold, we focused the conditional Q-Q plots on SNPs with nominal –log_10_(p) < 7.3 (corresponding to p > 5x10^-8^). We controlled for spurious enrichment by calculating all conditional Q-Q plots after random pruning averaged over 500 iterations. At each iteration, one SNP in every LD block (defined by an r^2^ >0.1) was randomly selected and the empirical cdfs were computed using the corresponding p-values.

## Detection of SNPs using conditional and conjunctional FDR

The FDR can be interpreted as the probability that a SNP is null given that its p-value is as small as or smaller than its observed p-value. The conditional FDR (condFDR) is an extension of the standard FDR, which incorporates information from GWAS summary statistics of a second phenotype to adjust its significance level. The condFDR is defined as the probability that a SNP is null in the first phenotype given that the p-values in the first and second phenotypes are as small as or smaller than the observed ones. It is important to note that ranking SNPs by the standard FDR or by p-values gives the same ordering of SNPs. In contrast, ranking SNPs by condFDR will reorder SNPs when the primary and secondary phenotypes are genetically related.

To identify SNPs that are associated with *both* phenotypes, we used conjunctional FDR (conjFDR)[9, 10], employing an overall FDR threshold of 0.05 according to the standard FDR approach[11]. The conjunctional FDR (conjFDR) is defined as the posterior probability that a SNP is null for either phenotype or both simultaneously, given that its p-values for association with both phenotypes are as small as or smaller than the observed p-values[9, 12-15]. A conservative estimate of the conjFDR is obtained by the maximum condFDR for a given SNP after repeating the condFDR procedure for both traits and inverting their roles[16]. Given that complex correlations in regions with intricate LD can bias FDR estimation[17], we excluded SNPs in the hg19 location chromosome 6: 25119106-33854733, hg19 location chromosome 8: 7242715-12483982 and hg19 location chromosome 17: 40000000 -47000000, before fitting the FDR models. Effect size (z-scores) of SNPs were obtained from the original summary statistics (see original publications for how they were calculated[18-28]). P-values were corrected for inflation using a genomic inflation control procedure[9].

## Genomic loci definition

We defined independent genomic loci using the FUMA, an online tool for functional mapping of genetic variants ([Functional Mapping and Annotation of Genome-wide association studies (ctglab.nl)](https://fuma.ctglab.nl/))[29]. Summary statistics from the GWASs on ASD, INT and EDU risk factors were used as input for FUMA. First, *independent* *significant SNPs* were identified as SNPs with condFDR < 0.01 and independent from each other at LD r^2^ < 0.6. Secondly, *lead SNPs* were identified by retaining those independent significant SNPs that were independent from each other at r^2^ < 0.1. Next, *distinct genomic loci* were identified by merging physically overlapping lead SNPs (LD blocks < 250 kb apart). Borders of the genomic loci were determined by identifying all SNPs in LD (r^2^ ≧ 0.6) with one of the independent significant SNPs in the locus. The region containing all of these *candidate SNPs* was regarded as a single independent genomic locus. All LD information was calculated from the 1000 Genomes Project reference panel [30].

# Functional annotation (FUMA)

We used FUMA[29], an online annotation platform (<http://fuma.ctglab.nl/>) that offers two analytic processes called “SNP2GENE” for gene mapping and “GENE2FUNC” for obtaining insight into putative biological mechanisms of prioritized genes.

FUMA SNP2GENE: this process was used to functionally annotate all candidate SNPs in the genomic loci with a condFDR cor conjFDR value<0.10 having an r^2^≧0.6 with one of the independent significant SNPs. SNPs were annotated with Combined Annotation Dependent Depletion (CADD) scores [32], RegulomeDB [33] scores, and chromatin states [34, 35] (see below). We conducted gene-set analysis to evaluate whether the genes mapped to the shared loci were overrepresented via FUMA[29]. We used Bonferroni-adjusted p-value threshold of 0.05 to correct for multiple comparisons.

The CADD score is a deleterious score of variants computed by integrating 63 functional annotations[32]. The higher the score, the more deleterious. A CADD score above 12.37 is the threshold to be potentially pathogenic[32]. The RegulomeDB score is a categorical score to guide interpretation of regulatory variants[33]. It is based on information from eQTLs and chromatin marks, ranging from 1a to 7 with lower scores indicating a higher likelihood of having a regulatory function. Scores are as follows: 1a=eQTL + Transcription Factor (TF) binding + matched TF motif + matched DNase Footprint + DNase peak; 1b=eQTL + TF binding + any motif + DNase Footprint + DNase peak; 1c=eQTL + TF binding + matched TF motif + DNase peak; 1d=eQTL + TF binding + any motif + DNase peak; 1e=eQTL + TF binding + matched TF motif; 1f=eQTL + TF binding / DNase peak; 2a=TF binding + matched TF motif + matched DNase Footprint + DNase peak; 2b=TF binding + any motif + DNase Footprint + DNase peak; 2c=TF binding + matched TF motif + DNase peak; 3a=TF binding + any motif + DNase peak; 3b=TF binding + matched TF motif; 4=TF binding + DNase peak; 5=TF binding or DNase peak; 6=other; 7=Not available[33].

The chromatin state represents the accessibility of genomic regions (every 200bp) with 15 categorical states predicted by a hidden Markov model based on 5 chromatin marks for 127 epigenomes in the Roadmap Epigenomics Project[35]. A lower state indicates increased accessibility, with states 1-7 referring to open chromatin states. We annotated the minimum chromatin state across tissues to SNPs. The 15-core chromatin states as suggested by Roadmap are as follows: 1=Active Transcription Start Site (TSS); 2=Flanking Active TSS; 3=Transcription at gene 5’ and 3’; 4=Strong transcription; 5= Weak Transcription; 6=Genic enhancers; 7=Enhancers; 8=Zinc finger genes & repeats; 9=Heterochromatic; 10=Bivalent/Poised TSS; 11=Flanking Bivalent/Poised TSS/Enh; 12=Bivalent Enhancer; 13=Repressed PolyComb; 14=Weak Repressed PolyComb; 15=Quiescent/Low.

We also used FUMA to link candidate and lead SNPs to genes using either of three gene-mapping strategies: 1) positional mapping to link SNPs to genes based on their physical proximity (i.e., within a 10kb window), 2) expression quantitative trait locus (eQTL) mapping to match cis-eQTL SNPs to genes whose expression is associated with allelic variation at the SNP level, and 3) chromatin interaction mapping to link SNPs to genes based on three-dimensional DNA–DNA interactions between each SNP’s genomic region and nearby or distant genes, as used in a recent GWAS from our group[36]. We considered eleven eQTL databases in FUMA which include eQTL information from several human tissue types including multiple brain regions (http://fuma.ctglab.nl/tutorial#eQTLs). The eQTL analyses were corrected for multiple comparisons using an FDR threshold of 0.05. FUMA includes Hi-C data of over 21 tissue/cell types including human brain tissue (https://fuma.ctglab.nl/tutorial#chromatin-interactions). We used an FDR of 1 x 10^-6^ to define significant chromatin interactions, in line with recommendations[37]. Analyses were corrected for multiple comparisons.

FUMA GENE2FUNC: To obtain insight into the function of the mapped gene sets, we used GENE2FUNC which offer an analytical strategy to provide information on how each gene in a gene set is expressed in different tissues, as well as on whether the gene set is a differentially expressed in various tissues and or wheter the gene set is overrepresented (enriched) in other gene sets, including pathways of biological function and other GWAS traits. The GENE2FUNC provides information only about pathways that are significantly associated with the investigated gene set, and not about non-significant pathways.

### Supplementary References

1. Rødevand, L., et al., *Polygenic overlap and shared genetic loci between loneliness, severe mental disorders, and cardiovascular disease risk factors suggest shared molecular mechanisms.* Transl Psychiatry, 2021. **11**(1): p. 3.

2. Frei, O., et al., *Bivariate causal mixture model quantifies polygenic overlap between complex traits beyond genetic correlation.* Nature communications, 2019. **10**(1): p. 2417-2417.

3. Holland, D., et al., *Beyond SNP Heritability: Polygenicity and Discoverability of Phenotypes Estimated with a Univariate Gaussian Mixture Model.* bioRxiv, 2019: p. 133132.

4. Bulik-Sullivan, B.K., et al., *LD Score regression distinguishes confounding from polygenicity in genome-wide association studies.* Nature Genetics, 2015. **47**(3): p. 291-295.

5. Benjamini, Y. and Y. Hochberg, *Controlling the False Discovery Rate: A Practical and Powerful Approach to Multiple Testing*, in *Journal of the Royal Statistical Society. Series B (Methodological)*. 1995, Blackwell Publishing. p. 289-300.

6. Efron, B., *Size, power and false discovery rates.* The Annals of Statistics, 2007. **35**(4): p. 1351–1377.

7. Purcell, S., et al., *PLINK: a tool set for whole-genome association and population-based linkage analyses.* Am J Hum Genet, 2007. **81**(3): p. 559-75.

8. Schweder, T. and E. Spjotvoll, *Plots of P-Values to Evaluate Many Tests Simultaneously.* Biometrika, 1982. **69**(3): p. 493-502.

9. Andreassen, O.A., et al., *Improved detection of common variants associated with schizophrenia by leveraging pleiotropy with cardiovascular-disease risk factors.* Am J Hum Genet, 2013. **92**(2): p. 197-209.

10. Schork, A.J., et al., *New statistical approaches exploit the polygenic architecture of schizophrenia--implications for the underlying neurobiology.* Curr Opin Neurobiol, 2016. **36**: p. 89-98.

11. Smeland, O.B., et al., *Discovery of shared genomic loci using the conditional false discovery rate approach.* Human Genetics, 2020. **139**(1): p. 85-94.

12. Andreassen, O.A., et al., *Improved detection of common variants associated with schizophrenia and bipolar disorder using pleiotropy-informed conditional false discovery rate.* PLoS Genet, 2013. **9**(4): p. e1003455.

13. Andreassen, O.A., et al., *Genetic pleiotropy between multiple sclerosis and schizophrenia but not bipolar disorder: differential involvement of immune-related gene loci.* Molecular Psychiatry, 2015. **20**(2): p. 207-214.

14. Andreassen, O.A., W.K. Thompson, and A.M. Dale, *Boosting the power of schizophrenia genetics by leveraging new statistical tools.* Schizophr Bull, 2014. **40**(1): p. 13-7.

15. Andreassen, O.A., et al., *Abundant genetic overlap between blood lipids and immune-mediated diseases indicates shared molecular genetic mechanisms.* PLoS One, 2015. **10**(4): p. e0123057.

16. Nichols, T., et al., *Valid conjunction inference with the minimum statistic.* Neuroimage, 2005. **25**(3): p. 653-60.

17. Schwartzman, A. and X. Lin, *The effect of correlation in false discovery rate estimation.* Biometrika, 2011. **98**(1): p. 199-214.

18. Schizophrenia Working Group of the Psychiatric Genomics Consortium, *Biological insights from 108 schizophrenia-associated genetic loci.* Nature, 2014. **511**(7510): p. 421-7.

19. Wray, N.R., et al., *Genome-wide association analyses identify 44 risk variants and refine the genetic architecture of major depression.* Nat Genet, 2018. **50**(5): p. 668-681.

20. Stahl, E.A., et al., *Genome-wide association study identifies 30 loci associated with bipolar disorder.* Nature Genetics, 2019. **51**(5): p. 793-803.

21. Locke, A.E., et al., *Genetic studies of body mass index yield new insights for obesity biology.* Nature, 2015. **518**(7538): p. 197-206.

22. Ehret, G.B., et al., *Genetic variants in novel pathways influence blood pressure and cardiovascular disease risk.* Nature, 2011. **478**(7367): p. 103-9.

23. Willer, C.J., et al., *Discovery and refinement of loci associated with lipid levels.* Nat Genet, 2013. **45**(11): p. 1274-1283.

24. Nikpay, M., et al., *A comprehensive 1,000 Genomes-based genome-wide association meta-analysis of coronary artery disease.* Nat Genet, 2015. **47**(10): p. 1121-1130.

25. Scott, R.A., et al., *An Expanded Genome-Wide Association Study of Type 2 Diabetes in Europeans.* Diabetes, 2017. **66**(11): p. 2888-2902.

26. Tobacco and C. Genetics, *Genome-wide meta-analyses identify multiple loci associated with smoking behavior.* Nature genetics, 2010. **42**(5): p. 441-447.

27. Day, F.R., K.K. Ong, and J.R.B. Perry, *Elucidating the genetic basis of social interaction and isolation.* Nature Communications, 2018. **9**(1): p. 2457.

28. Turcot, V., et al., *Protein-altering variants associated with body mass index implicate pathways that control energy intake and expenditure in obesity.* Nat Genet, 2018. **50**(1): p. 26-41.

29. Watanabe, K., et al., *Functional mapping and annotation of genetic associations with FUMA.* Nat Commun, 2017. **8**(1): p. 1826.

30. The 1000 Genomes Project Consortium, *A global reference for human genetic variation.* Nature, 2015. **526**(7571): p. 68-74.

31. Bulik-Sullivan, B., et al., *An atlas of genetic correlations across human diseases and traits.* Nature Genetics, 2015. **47**(11): p. 1236-1241.

32. Kircher, M., et al., *A general framework for estimating the relative pathogenicity of human genetic variants.* Nat Genet, 2014. **46**(3): p. 310-5.

33. Boyle, A.P., et al., *Annotation of functional variation in personal genomes using RegulomeDB.* Genome Res, 2012. **22**(9): p. 1790-7.

34. Zhu, Z., et al., *Integration of summary data from GWAS and eQTL studies predicts complex trait gene targets.* Nat Genet, 2016. **48**(5): p. 481-7.

35. Kundaje, A., et al., *Integrative analysis of 111 reference human epigenomes.* Nature, 2015. **518**(7539): p. 317-330.

36. Smeland, O.B., et al., *Genome-wide Association Analysis of Parkinson’s Disease and Schizophrenia Reveals Shared Genetic Architecture and Identifies Novel Risk Loci.* Biological Psychiatry.

37. Schmitt, A.D., et al., *A Compendium of Chromatin Contact Maps Reveals Spatially Active Regions in the Human Genome.* Cell Rep, 2016. **17**(8): p. 2042-2059.

1. [↑](#footnote-ref-1)
