# Supplementary material for: Bidirectional genetic overlap between autism spectrum disorder and cognitive traits
Source: Transl Psychiatry. 2023 Sep 14;13:295. doi: 10.1038/s41398-023-02563-7 (PMC10502136; doi:10.1038/s41398-023-02563-7)
Supplement: Supplementary file 2 — Supplementary Results, Figure S1-S9 and Table 16 [file 41398_2023_2563_MOESM2_ESM.docx]

**Supplementary Figures**

Content

[Genetic overlap from MiXer Analysis 2](#_Toc134022189)

[Figure S1: Conditional Q-Q plots and log - likelihood plots 2](#_Toc134022190)

[a) ASD and EDU 2](#_Toc134022191)

[b) ASD and INT 2](#_Toc134022192)

[Locus mapping 3](#_Toc134022193)

[Figure S2: Manhattan plots showing genetic loci from condFDR analyses 3](#_Toc134022194)

[S2 a) ASD and EDU 3](#_Toc134022195)

[S2 b) ASD and INT 4](#_Toc134022196)

[FUMA GENE2FUNC, total gene sets irrespective of effect direction 5](#_Toc134022197)

[Figure S3:FUMA for ASD and EDU 5](#_Toc134022198)

[Heatmap showing how each gene is genes expressed in different tissues 5](#_Toc134022199)

[b) Histogram showing differentially expressed gene set (DEG) in 54 tissue types 6](#_Toc134022200)

[c) Histogram showing brain developmental stages and differentially expressed gene set (DEG) 7](#_Toc134022201)

[Figure S4: FUMA for ASD and INT 8](#_Toc134022202)

[a) Heatmap showing how each gene is genes expressed in different tissues 8](#_Toc134022203)

[b) Histogram showing differentially expressed gene set (DEGs) in 54 tissue types 9](#_Toc134022204)

[c) Histogram showing brain developmental stages and differentially expressed gene set (DEG) 10](#_Toc134022205)

[FUMA GENE2FUNC; concordant genes 11](#_Toc134022206)

[Figure S5 FUMA, concordant gene set, ASD and EDU 11](#_Toc134022207)

[a) Heatmap showing how each gene is genes expressed in different tissues 11](#_Toc134022208)

[b) Histogram showing differentially expressed gene set in 54 tissue types 13](#_Toc134022209)

[c) Histogram showing brain developmental and differentially expressed gene set (DEG) 14](#_Toc134022210)

[Figure S6: FUMA concordant gene set, ASD and INT 15](#_Toc134022211)

[a) Heatmap showing how each gene is genes expressed in different tissues 15](#_Toc134022212)

[b) Histogram showing differentially expressed gene set in 54 tissue types 16](#_Toc134022213)

[c) Brain developmental stages 17](#_Toc134022214)

[FUMA GENE2FUNC discordant genes 18](#_Toc134022215)

[Figure S7: FUMA discordant gene set, ASD and EDU 18](#_Toc134022216)

[Heatmap 18](#_Toc134022217)

[Differentially expressed gene sets in tissue types 19](#_Toc134022218)

[Figure S8: FUMA discordant genes, ASD, INT 20](#_Toc134022219)

[Heatmap showing tissue expression in 54 tissue types 20](#_Toc134022220)

[Supplementary figure S8 a) Histogram, ASD and INT , discordant genes, differentially expressed genes in 54 tissues 20](#_Toc134022221)

[Figure S9: Credible genes, enrichment in other GWAS traits: 21](#_Toc134022222)

[Concordant 21](#_Toc134022223)

[Discordant 21](#_Toc134022224)

[Table S16. Credible genes associated with ASD and cognitive traits 22](#_Toc134022225)

# Genetic overlap from MiXer Analysis

## Figure S1: Conditional Q-Q plots and log - likelihood plots

### a) ASD and EDU

### b) ASD and INT

**Supplementary Figure S1.** Conditional QQ plots of observed versus expected -log10 p-values in the primary trait as a function of significance of association with the secondary trait at the level of p ≤ 0.1 (orange lines), p ≤ 0.01 (green lines) and p ≤ 0.001 (red lines). Blue lines indicate all SNPs. Dotted lines indicate model predictions for each stratum. Black dotted line is the expected Q-Q plot under the null hypothesis (no SNPs associated with the phenotype). Likelihood cost: Log-likelihood of the bivariate fit as a function of � parameter. The remaining parameters of the model were constrained to their fitted values

# Locus mapping

## Figure S2: Manhattan plots showing genetic loci from condFDR analyses

| S2 a) ASD and EDUSNPs associated with ASD conditional on EDU |
| --- |


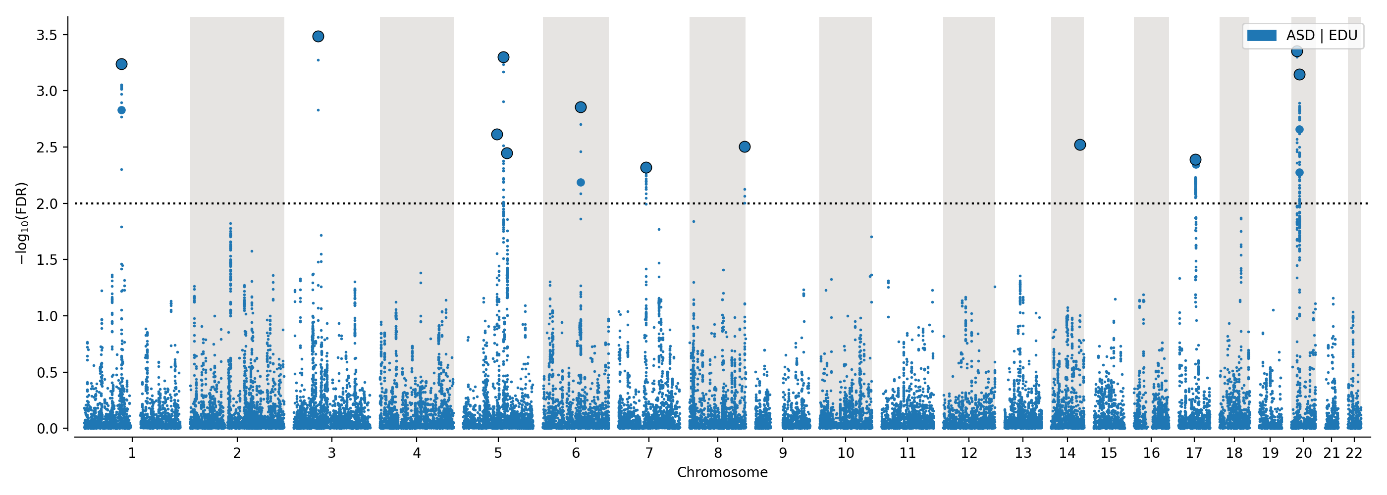


#### SNPs associated with EDU conditional on ASD


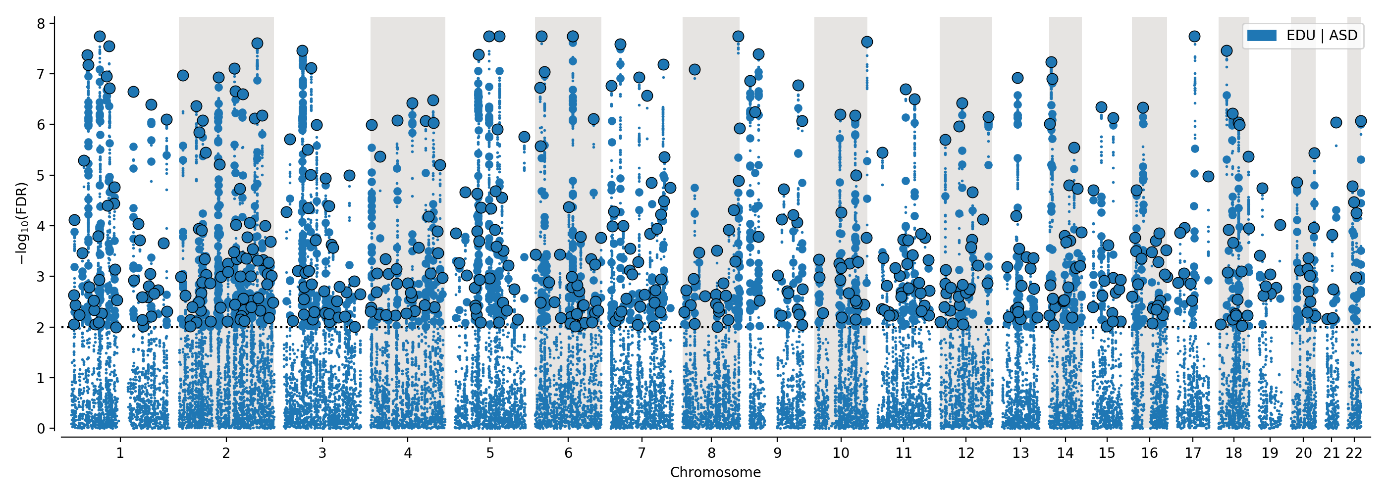


**Supplementary Figure S2 a).** Common genetic variants jointly associated with ASD and EDU at conditional false discovery rate (condFDR) < 0.05. The dotted horizontal line represents the threshold for significant shared associations (condFDR < 0.05, i.e., −log10 (conjFDR) > 2.0). Independent lead SNPs are encircled in black.

### S2 b) ASD and INT

#### SNPs associated with ASD conditional on INT


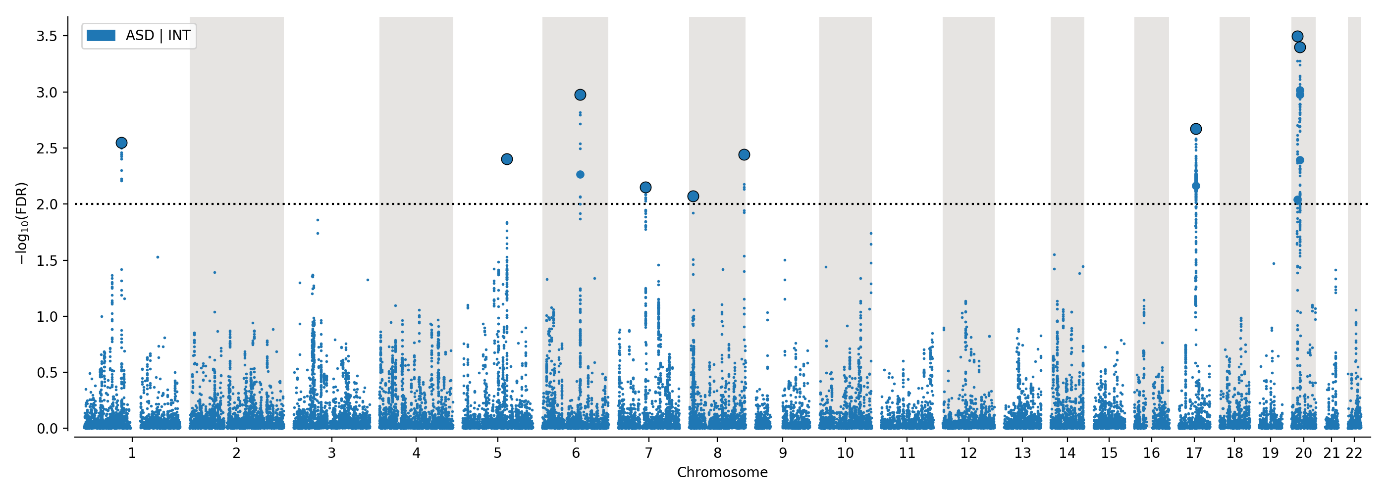


#### SNPs associated with INT conditional on ASD

**
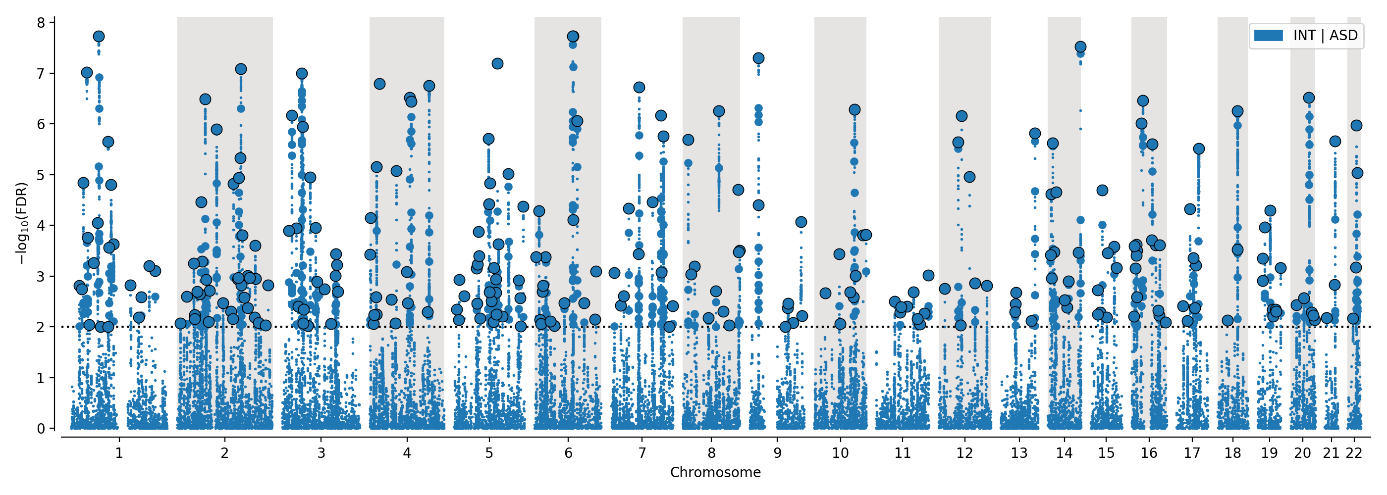
**

**Supplementary Figure S2 b)**. Common genetic variants jointly associated with ASD and INT at conditional false discovery rate (condFDR) < 0.05. The dotted horizontal line represents the threshold for significant shared associations (condFDR < 0.05, i.e., −log10 (conjFDR) > 2.0). Independent lead SNPs are encircled in black.

# FUMA GENE2FUNC, total gene sets irrespective of effect direction

## Figure S3:FUMA for ASD and EDU

### Heatmap showing how each gene is genes expressed in different tissues

**
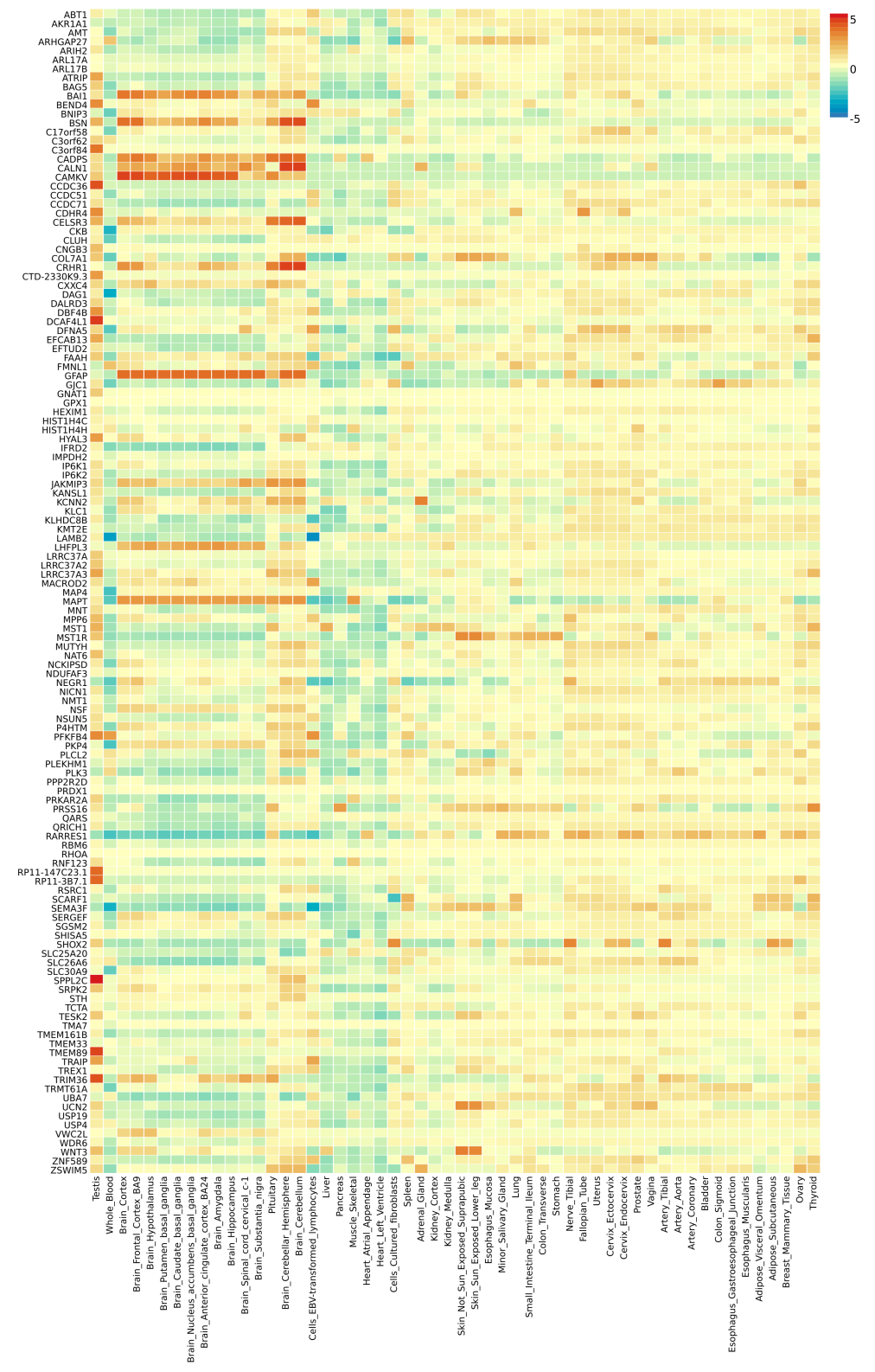
**

**Supplementary figure S3 a).** Heatmap showing expression in different tissues for each of the mapped gene in the gene set associated with ASD and EDU. The gene set includes both concordant and discordant genes

### b) Histogram showing differentially expressed gene set (DEG) in 54 tissue types


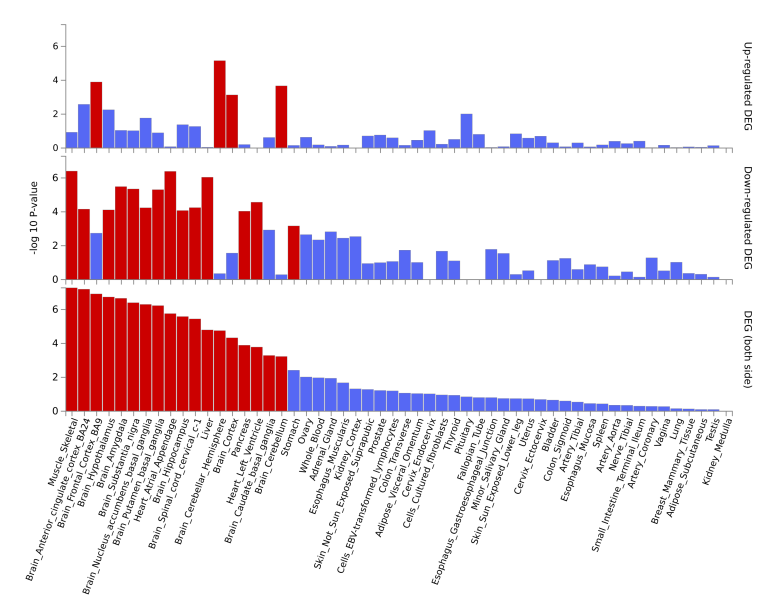


**Supplementary figure S3 b).** Histogram showing whether the gene set associated with ASD and EDU is a differentially expressed gene set (DEG) in different tissues. Red bars indicate statistical significance after correcting for multiple testing. Histogram showing tissue specificity based on GTEx v8 54 tissue types.

### c) Histogram showing b**rain** developmental stages and differentially expressed gene set (DEG)


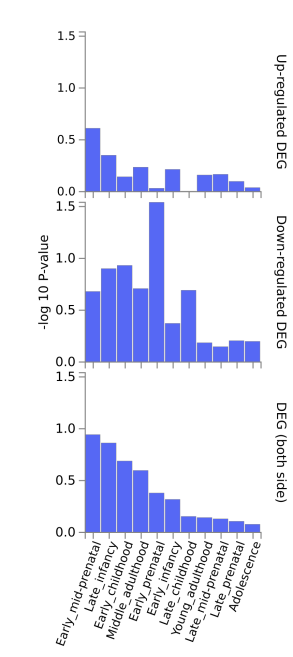


**Supplementary figure S3 c).** Histogram showing whether the gene set associated with ASD and EDU is a differentially expressed gene set (DEGs) in different brain development stages based on BrainSpan 11 general developmental stages of brain samples.

## Figure S4: FUMA for ASD and INT

### a) Heatmap showing how each gene is genes expressed in different tissues


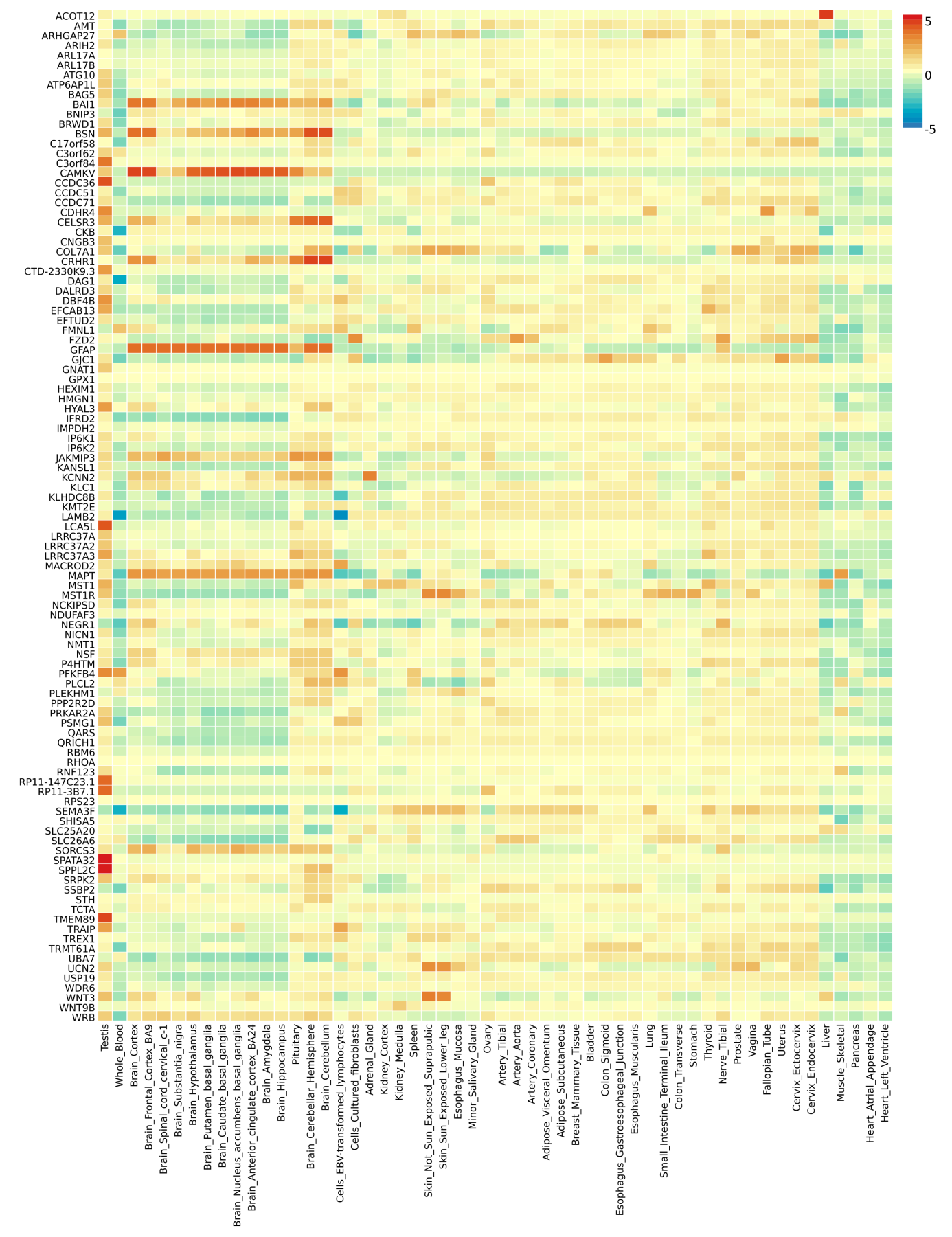


**Supplementary figure S4 a).** Heatmap showing expression or each of the mapped genes in the gene set associated with ASD and INT. The gene set includes both concordant and discordant genes

### b) Histogram showing differentially expressed gene set (DEGs) in 54 tissue types

###


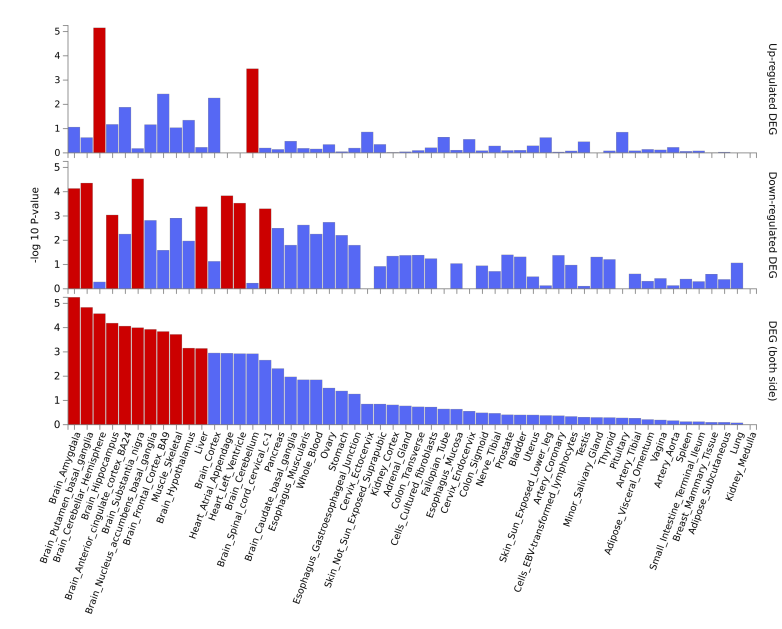


**Supplementary figure S4 b).** Histogram showing whether the gene set associated with ASD and INT is a differentially expressed gene set (DEG) in different tissues. Red bars indicate statistical significance after correcting for multiple testing. Histogram showing tissue specificity based on GTEx v8 54 tissue types

### c) Histogram showing brain developmental stages and differentially expressed gene set (DEG)


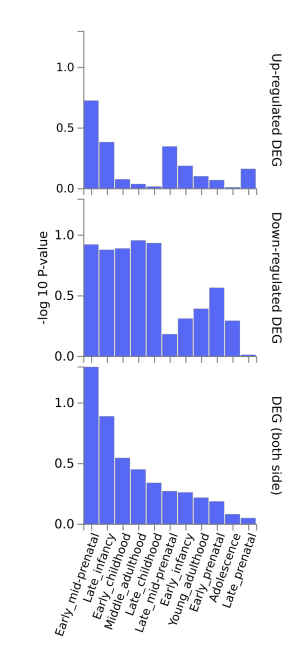


**Supplementary figure S4 c).** Histogram showing whether the gene set associated with ASD and INT is a differentially expressed gene set (DEGs) in different brain development stages based on BrainSpan 11 general developmental stages of brain samples.

# FUMA GENE2FUNC; concordant genes

## Figure S5 FUMA, concordant gene set, ASD and EDU

### a) Heatmap showing how each gene is genes expressed in different tissues


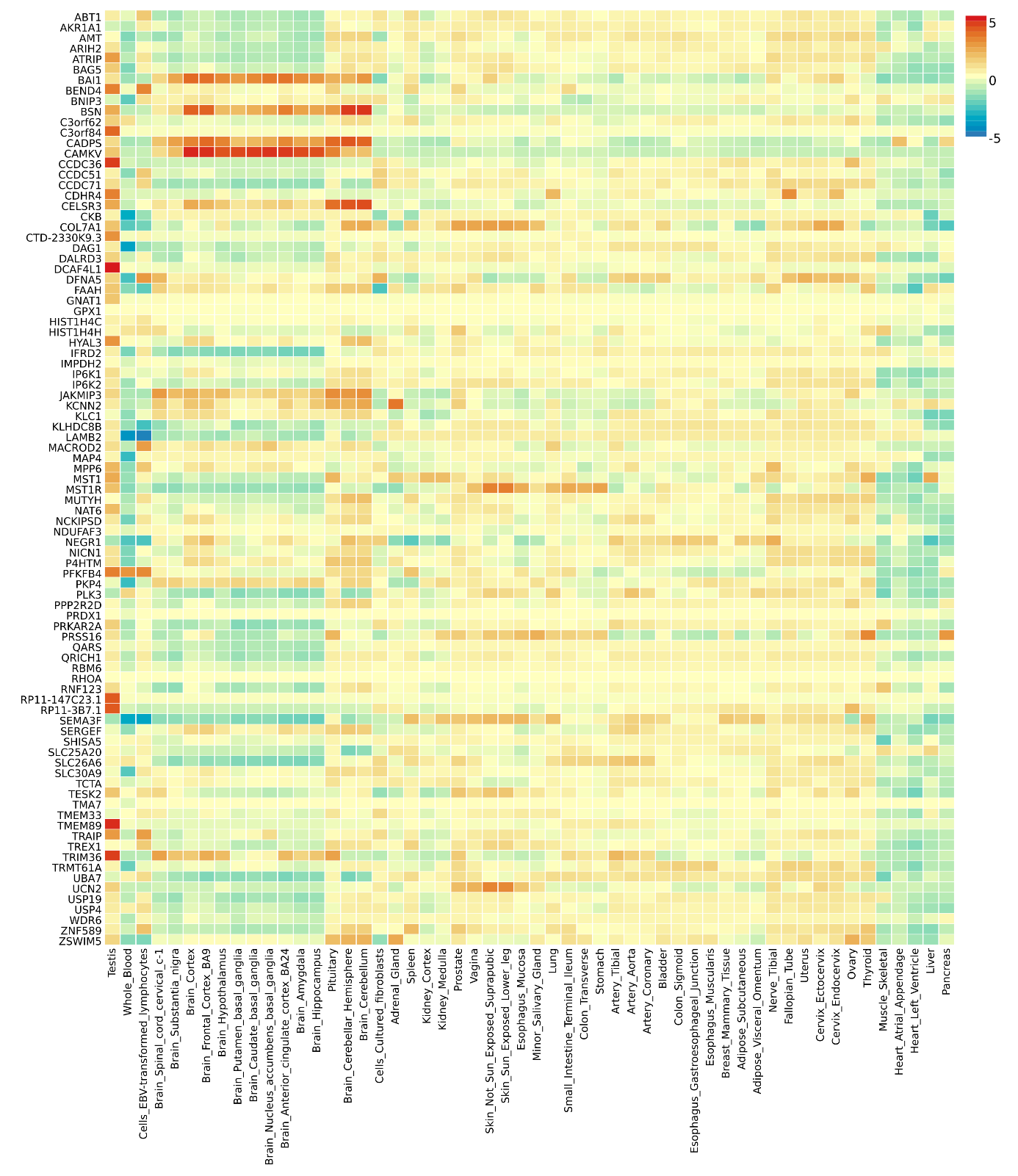


**Supplementary figure S5 a).** Heatmap showing expression for each of the mapped genes being concordantly associated with ASD and EDU.

### b) Histogram showing differentially expressed gene set in 54 tissue types


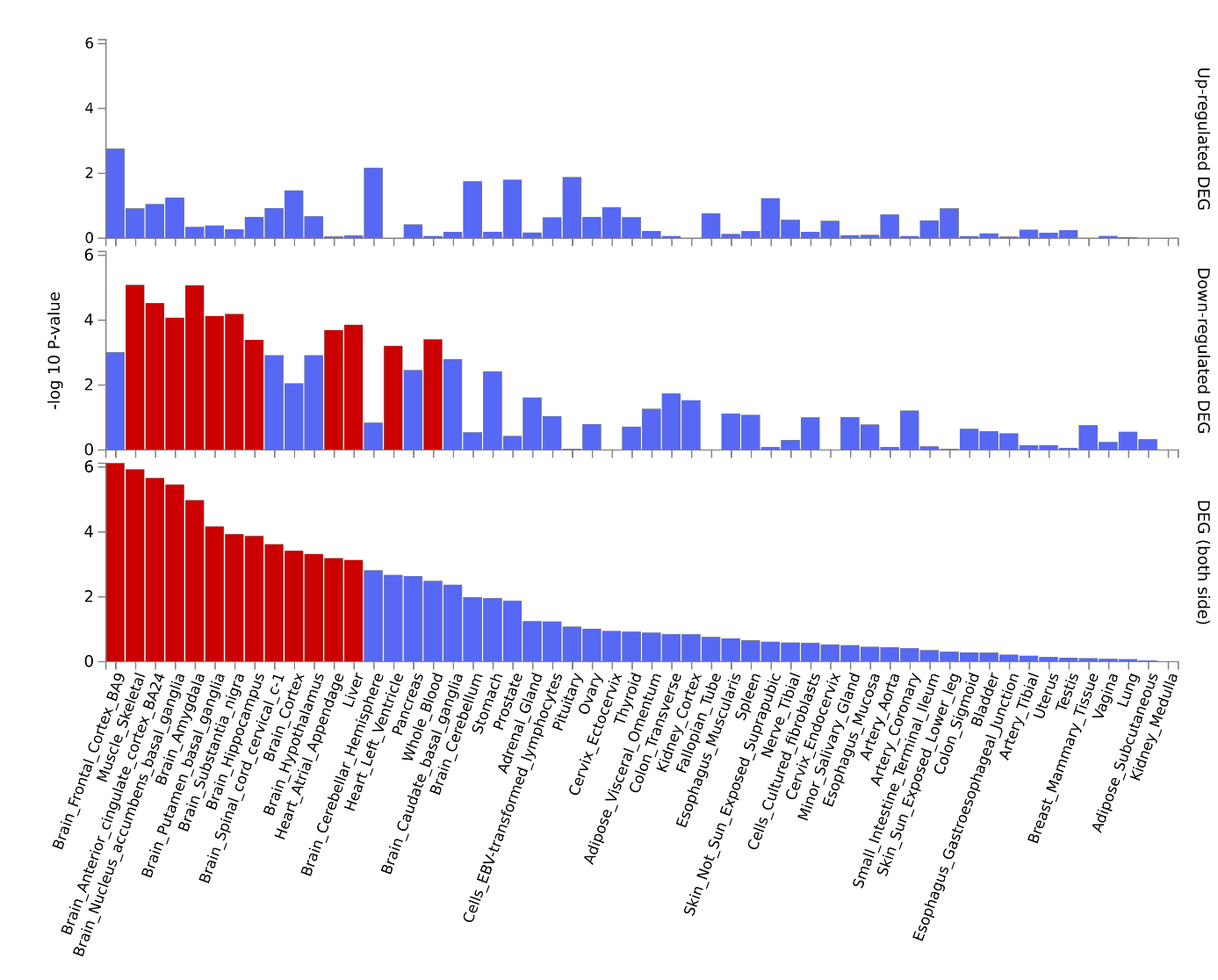


Supplementary figure S5 b). Histogram showing whether the gene set concordantly associated with ASD and EDU is a differential expression of gene set (DEG) in different tissues. Red bars indicate statistical significance after correcting for multiple testing. Histogram showing tissue specificity based on GTEx v8 54 tissue types.

### c) Histogram showing brain developmental and differentially expressed gene set (DEG)


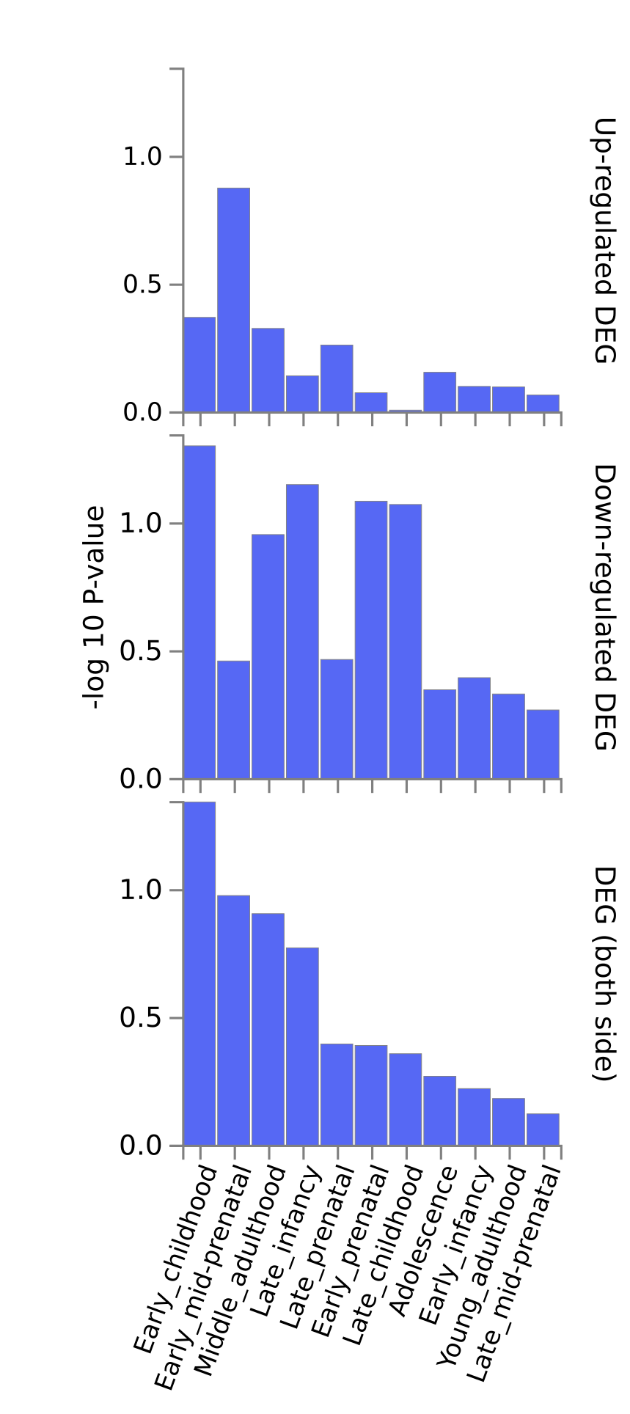


**Supplementary figure S5 c).** Histogram showing whether the gene set concordantly associated with ASD and EDU INT is a differentially expressed gene set (DEGs) in different brain development stages based on BrainSpan 11 general developmental stages of brain samples.

## Figure S6: FUMA concordant gene set, ASD and INT

### a) Heatmap showing how each gene is genes expressed in different tissues


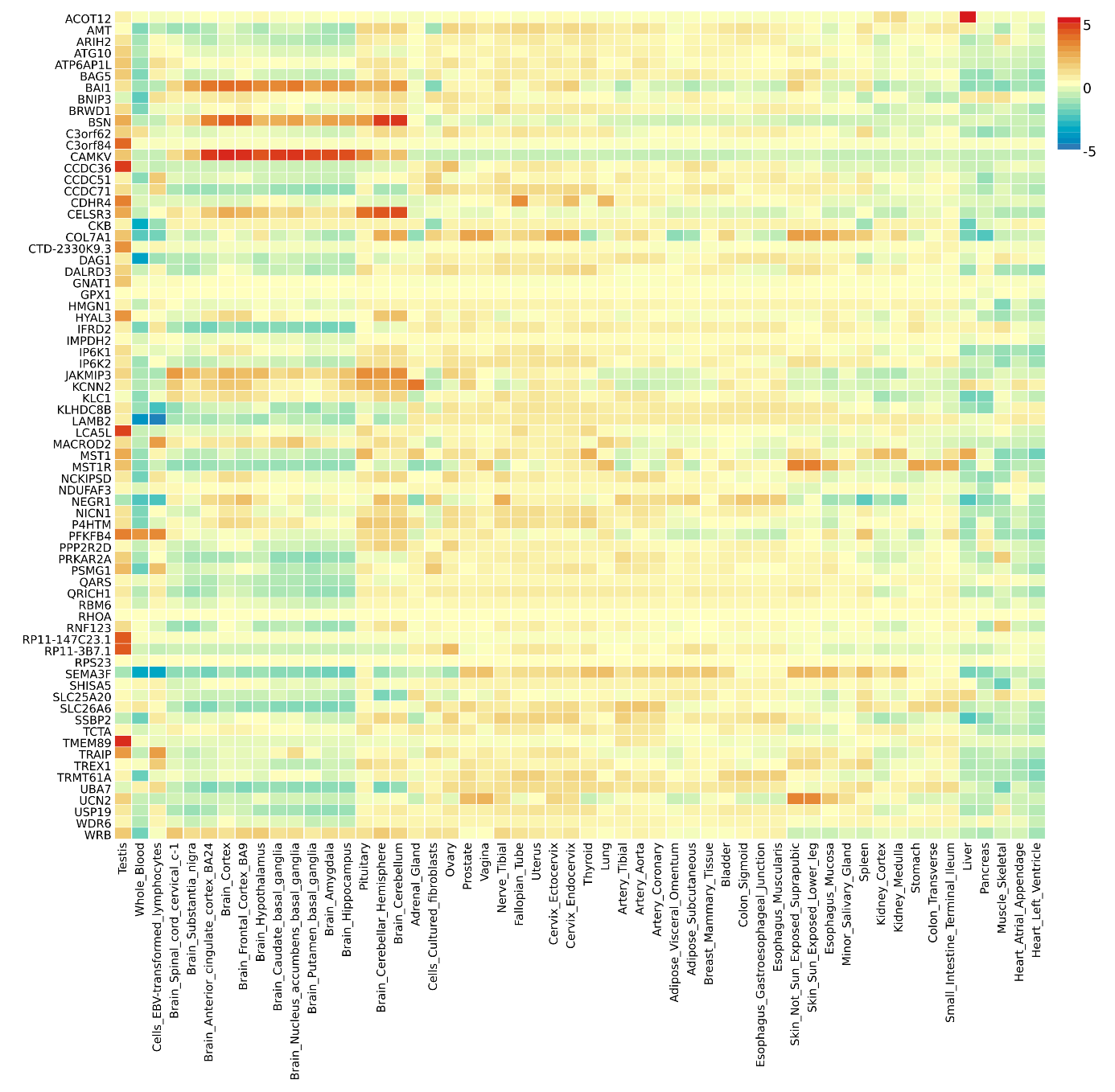


**Supplementary figure S6 a).** Heatmap showing expression for each of the mapped genes being concordantly associated with ASD and INT.

### b) Histogram showing differentially expressed gene set in 54 tissue types


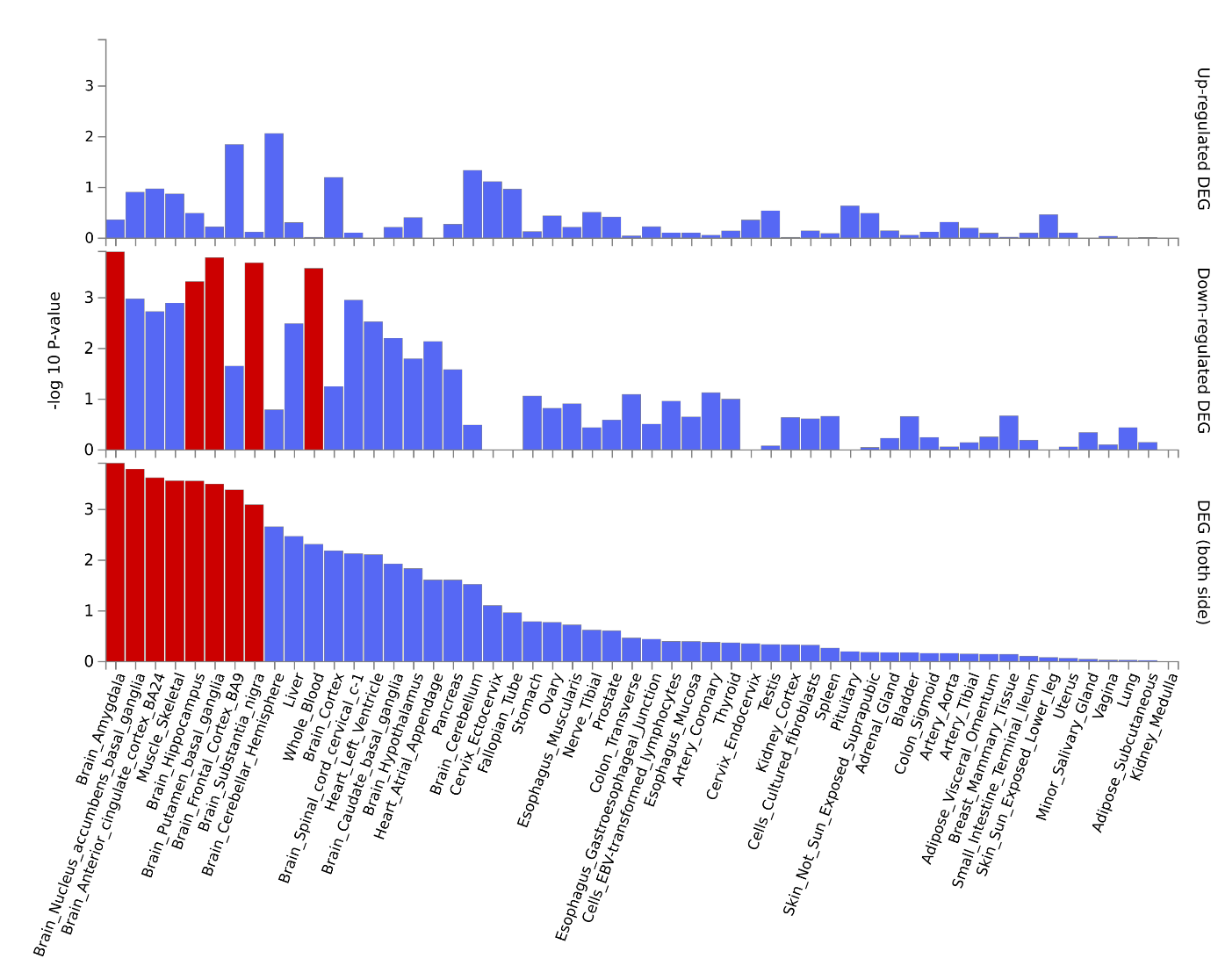


**Supplementary figure S6 b).** Differential expression of genes (DEG) by tissue for the genes annotated to concordant SNPs associated with ASD and INT. Red bars indicate statistical significance after correcting for multiple testing. Histogram showing tissue specificity based on GTEx v8 54 tissue types.

### c) Brain developmental stages


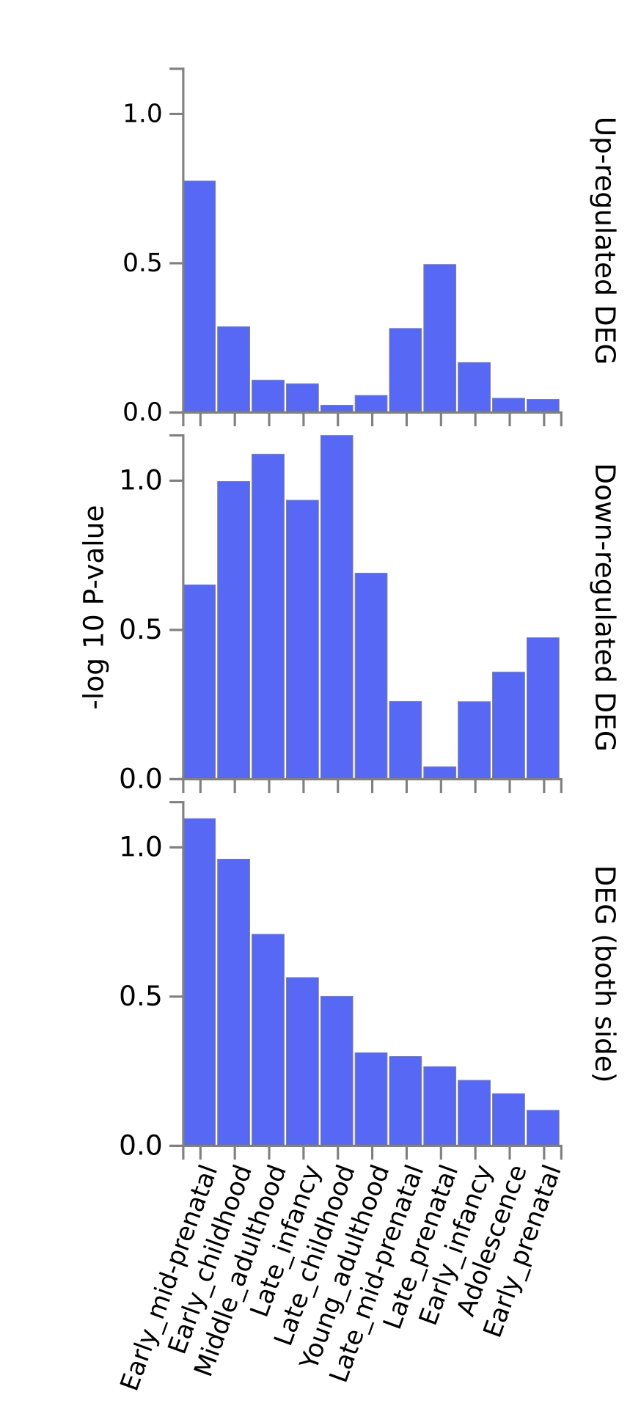


**Supplementary figure S5 c).** Differentially expressed genes (DEGs) in brain development stages for all concordant SNPs between ASD and INT. Based on BrainSpan 11 general developmental stages of brain samples.

# FUMA GENE2FUNC discordant genes

## Figure S7: FUMA discordant gene set, ASD and EDU

### Heatmap


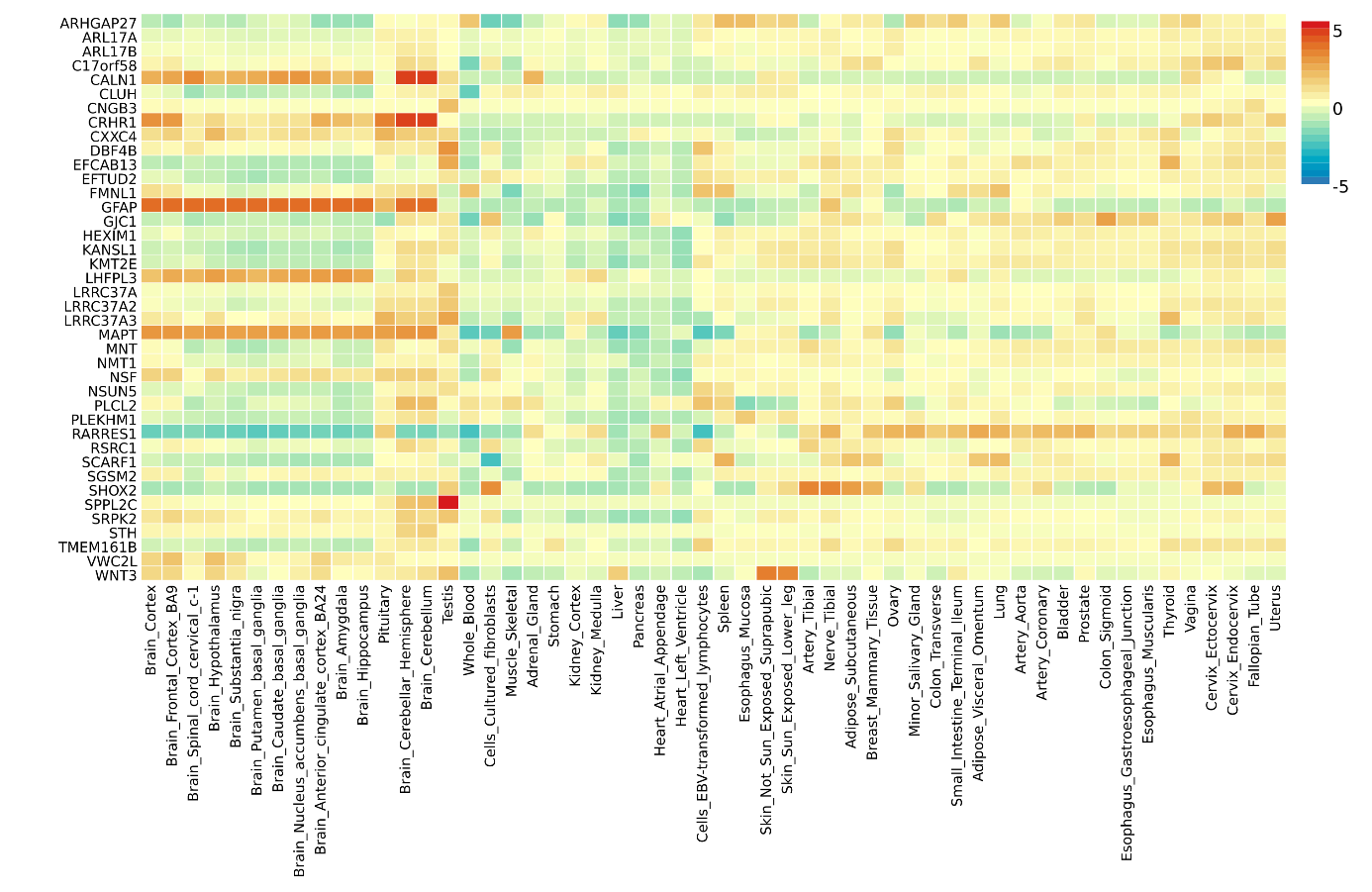


**Supplementary figure S7 a).** Heatmap showing tissue expression of discordant genes for ASD and EDU.

### Differentially expressed gene sets in tissue types


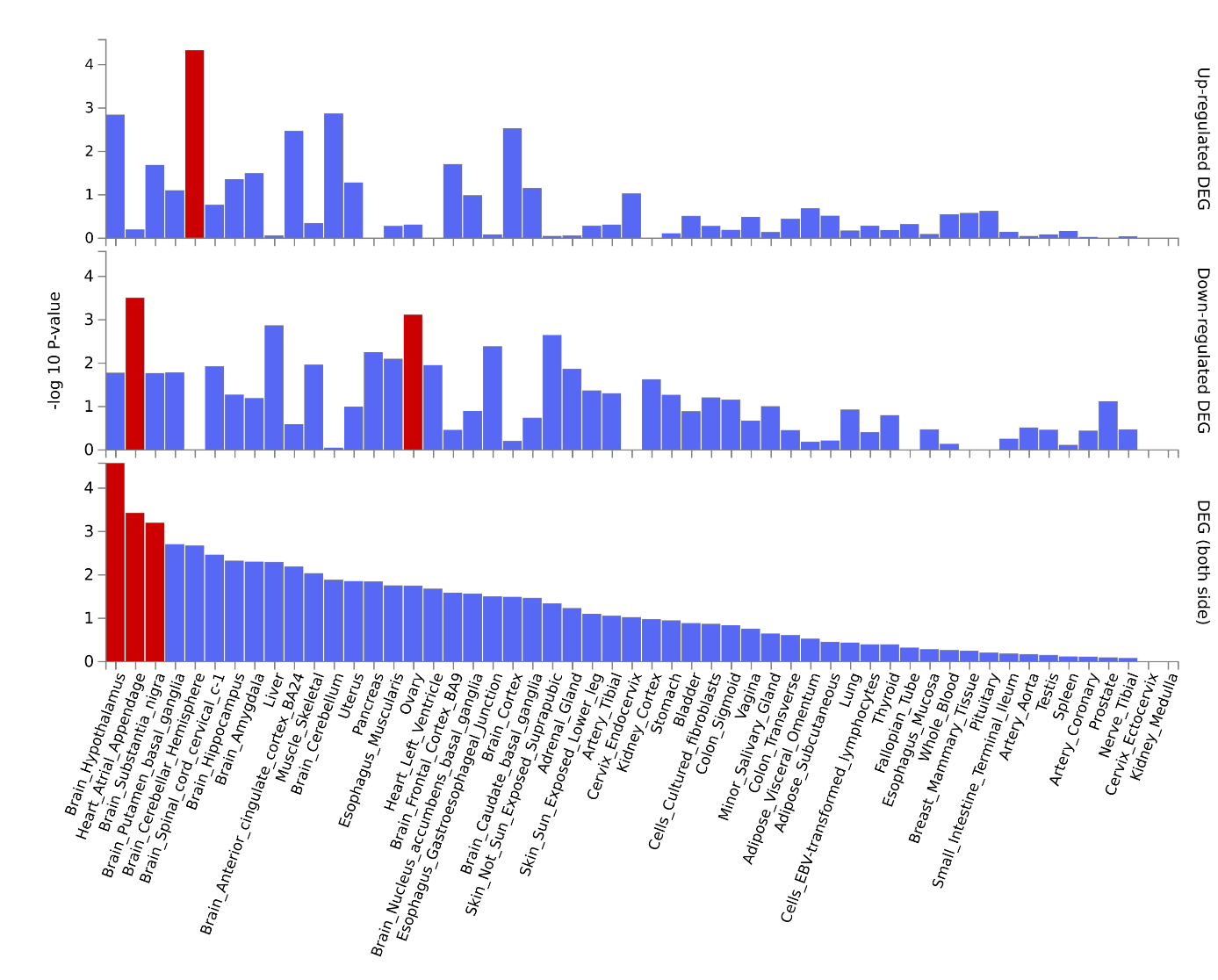


**Supplementary figure S7 b).** Differential expression of genes (DEG) by tissue for the genes annotated to discordant SNPs associated with ASD and EDU. Red bars indicate statistical significance after correcting for multiple testing. Histogram showing tissue specificity based on GTEx v8 54 tissue types.

## Figure S8: FUMA discordant genes, ASD, INT

### Heatmap showing tissue expression in 54 tissue types


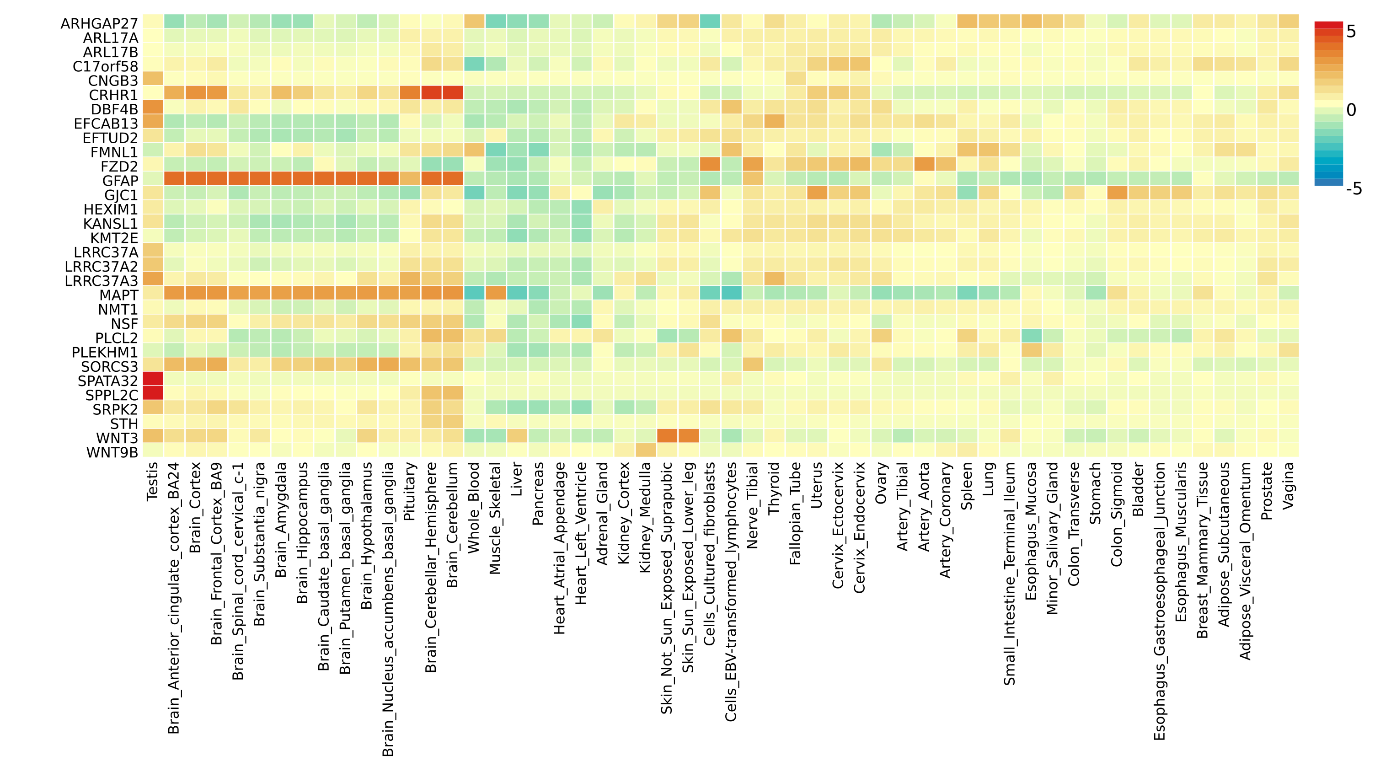


### Supplementary figure S8 a) Histogram, ASD and INT , discordant genes, differentially expressed genes in 54 tissues


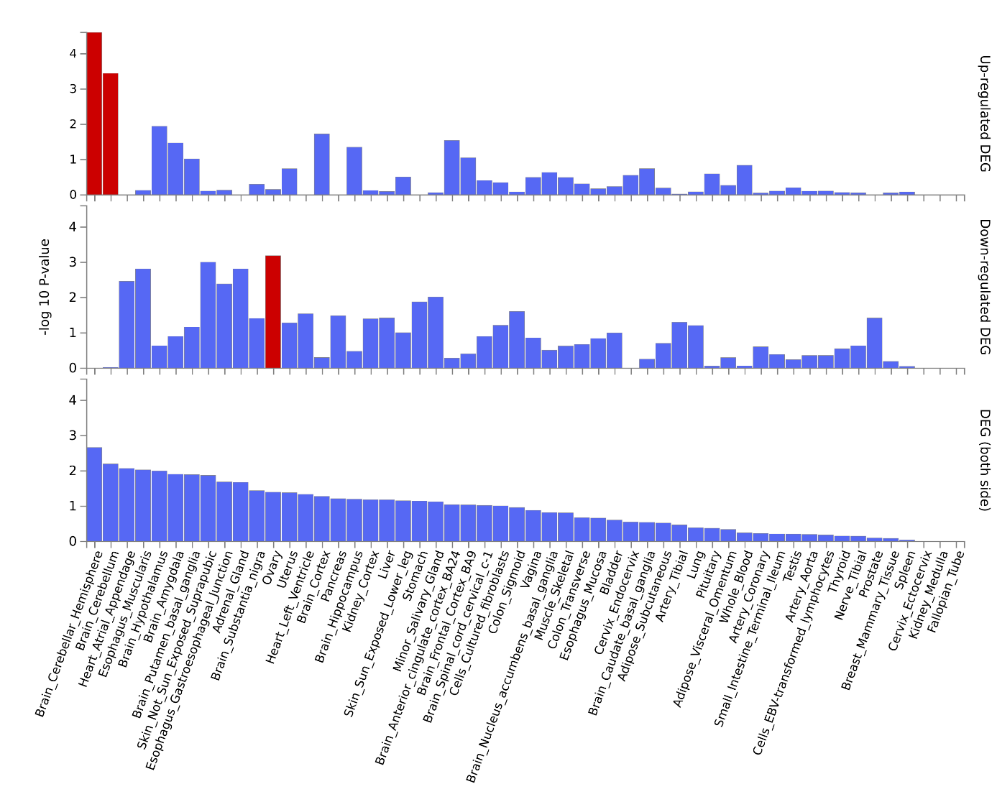


**Supplementary figure S8 b).** Differential expression of genes (DEG) by tissue for the genes annotated to disconcordant SNPs associated with ASD and INT. Red bars indicate statistical significance after correcting for multiple testing. Histogram showing tissue specificity based on GTEx v8 54 tissue types

# Figure S9: Credible genes, enrichment in other GWAS traits:

### Concordant


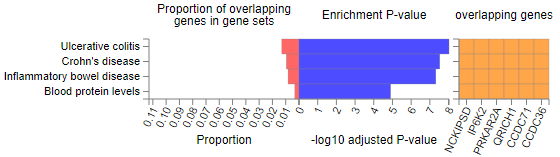


###

### Discordant


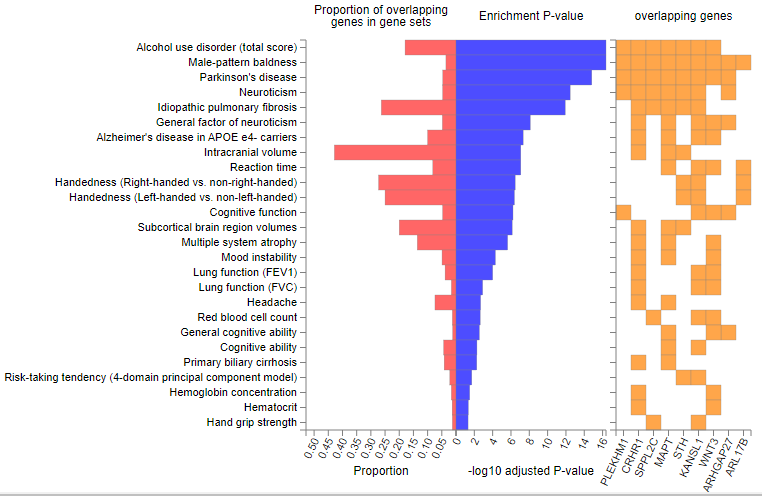


# Table S16. Credible genes associated with ASD and cognitive traits

Credible genes: mapped with genetic loci jointly associated with ASD and cognitive traits by three different gene mapping methods. Concordant: mapped for genetic loci associated with both ASD and good cognitive abilities. Discordant: mapped from loci associated with both ASD and cognitive difficulties.

| **Gene** | **Function** | **Associated diseases** | **Enriched in pathways** | |
| --- | --- | --- | --- | --- |
| **Concordant** |  |  | |  |
| NCKIPSD | Modulates neuronal synapses | Wiskott-Aldrich Syndrome with immune deficiency, eczema and bleeding | | Inflammatory bowel diseases (Chrohn’s and ulcerative colitis) and blood protein levels. |
| CCDC36 | Modulates neuronal synapses  Inositol phosphokinase family protein, cyclic AMP signaling | Wiskott-Aldrich Syndrome with immune deficiency, eczema and bleeding  Autoimmune disesase | |  |
| IP6K2 |  |  |  |  |
| PRKAR2A | Regulatory Protein Kinase, Cyclic AMP-Dependent | Nivelon-Mabille Syndrome (microcephaly, cerebellar hypoplasia and skeletal dysplasia) and [Carney Complex Variant](https://www.malacards.org/card/carney_complex_variant) (risk of tumors and skin pigmentation) | |  |
| QRICH1 | Response to endoplasmatic reticulum stress | QRICH1-neurodevelopmental disorder, [Cerebello-facio-dental Syndrome](https://www.malacards.org/card/cerebellofaciodental_syndrome) | |  |
| CCDC71 | Reproductive cell production | Male [infertility](https://www.malacards.org/card/infertility) | |  |
| **Discordant** |  |  | |  |
| MAPT | Tau–protein which may form [neurofibrillary tangles](https://en.wikipedia.org/wiki/Neurofibrillary_tangles) in the brain | Alzheimer's, Frontotemporal dementia, Pick's disease, cortico-basal degeneration and progressive supranuclear palsy | | Mental disease/traits: Alcohol use disorder, neuroticism, mood instability, chronic pain, headache and risk taking tendency  Neurodegenerative diseases/traits: Alzheimer’s disease, Parkinson’s disease, Multiple system atrophy, intracranial and subcortical volume  Somatic diseases /traits: male-pattern baldness, lung function, reaction time and handedness, primary biliary cirrhosis, sense of smell, hand grip strength, hemoglobin and hematocrit |
| CRHR1 | Corticotropin Releasing Hormone Receptor, modulates stress responses | [Anxiety](https://www.malacards.org/card/anxiety) and [Major Depressive Disorder](https://www.malacards.org/card/major_depressive_disorder). | |  |
| WNT3 | WNT family member 3 | Tetraamelia syndrome (no limbs) | |  |
| KANSL1 | Histone acetylation, regulates cell proliferation | Koolen-de Vries Syndrome (intellectual disability, congenital malformations, and behavioral features) | |  |
| ARL17B | GTP binding/energy transfer | Koolen-de Vries Syndrome (intellectual disability, congenital malformations, and behavioral features)  [Kanzaki](https://www.malacards.org/card/kanzaki_disease)/[Schindler disease](https://www.malacards.org/card/schindler_disease) (often developmental delay, seizures and behavioral problems) | |  |
| SPPL2C | Vesicular transport, acrosome |  |  |  |
| LRRC37A | Leucine rich membrane protein |  |  |  |
| ARHGAP27 | Rho GTPase Activating |  |  |  |
| PLEKHM1 | Regulates bone resorption | Osteopetrosis (bone disesase) | |  |
| STH | MAPTiT, Tau/dementia-related protein | Infections (Guinea worm and [Filarial Elephantiasis](https://www.malacards.org/card/filarial_elephantiasis)) | |  |

Credible genes: mapped with genetic loci jointly associated with ASD and cognitive traits by three different gene mapping methods. Concordant: mapped for genetic loci associated with both ASD and good cognitive abilities. Discordant: mapped from loci associated with both ASD and cognitive difficulties.
